# Responses of healthy young males to fine-particle exposure are modified by exercise habits: a panel study

**DOI:** 10.1186/s12940-018-0437-3

**Published:** 2018-12-13

**Authors:** Xi Chen, Wu Chen, Yanwen Wang, Yiqun Han, Tong Zhu

**Affiliations:** 1 State Key Joint Laboratory for Environmental Simulation and Pollution Control, College of Environmental Sciences and Engineering, Peking University, Beijing, 100871 China; 2 The Beijing Innovation Center for Engineering Science and Advanced Technology, Peking University, Beijing, 100871 China; 3 Center of Research and Innovation, Shenzhen Institute of Building Research Co., Ltd., Shenzhen, 518049 China

**Keywords:** PM_2.5_, Exercise habits, Airway inflammation, Arterial stiffness

## Abstract

**Background:**

Aerobic exercise benefits health but increases inhalation of fine particles (PM_2.5_) in ambient air. Acute cardiopulmonary responses to PM_2.5_ exposure in individuals with different exercise habits, especially in areas with severe air pollution, are not well understood.

**Methods:**

To examine acute cardiopulmonary responses to PM_2.5_ exposure modified by exercise habits, a panel of 20 healthy non-smoking male subjects, recruited in Beijing, China, completed seven visits. The exercise frequency per week and preferred exercise place were recorded using a baseline questionnaire to describe exercise habits. Fractional exhaled nitric oxide (FeNO), cytokines in exhaled breath condensate, blood pressure, and pulse-wave analysis (PWA) indices were measured during each visit as biomarkers of acute cardiopulmonary responses. The hourly average mass concentration of PM_2.5_ and black carbon (BC), and the number concentrations of ultrafine particles (UFP) and accumulation mode particles (AMP) were monitored throughout the follow-up period at an outdoor fixed monitoring station beginning 14 days prior to each visit. Linear mixed-effects models were used to evaluate the associations between acute changes in biomarker levels and exposure to PM_2.5_ and its constituents. The primary aim was to assess the modification of long-term exercise habits on these associations.

**Results:**

FeNO concentration, systolic blood pressure, ejection duration, aortic augmentation pressure, and aortic pressure index were positively associated with exposure to PM_2.5_ and its constituents. However, no associations with cytokine levels or diastolic blood pressure were observed. In a stratified analysis, we found that acute cardiopulmonary responses were modified by exercise habit. Specifically, the interquartile ranges (IQR) of increases in the 6–12-h moving average (MA) PM_2.5_ and AMP exposure were associated with 19–21% and 24–26% increases in FeNO, respectively, in subjects with high exercise frequency; these associations were significantly stronger than those in subjects with low exercise frequency. An IQR increase in 3–11-d MA AMP exposure was associated with a 10–26% increase in aortic augmentation pressure in subjects with low exercise frequency; this association was significantly stronger than that in subjects with high exercise frequency. An IQR increase in 9–13-d MA UFP exposure was associated with a 13–17% increase in aortic augmentation pressure in subjects who preferred outdoor exercise; this association was stronger than that in subjects who preferred indoor exercise.

**Conclusions:**

In highly polluted areas, frequent exercise might protect against PM_2.5_-associated arterial stiffness but exacerbate airway inflammation.

**Electronic supplementary material:**

The online version of this article (10.1186/s12940-018-0437-3) contains supplementary material, which is available to authorized users.

## Background

Short- and long-term exposure to fine particles (PM_2.5_, particulate matter with an aerodynamic diameter ≤ 2.5 μm) in ambient air is strongly linked to adverse effects on human health, ranging from subclinical changes in cardiopulmonary biomarkers to premature mortality and morbidity [1–3]. Pulmonary and systemic inflammation, oxidative stress, increased blood coagulation, autonomic and vascular imbalance, and endothelial dysfunction have been reported as mechanisms of the cardiopulmonary effects of PM_2.5_ [4–6]. Black carbon (BC), ultrafine particles (UFP), and accumulation mode particles (AMP) may contribute to the cardiopulmonary effects of PM_2.5_ exposure [4, 7–11].

Long-term moderate exercise improves endothelial function, adaptation of the cardio-pulmonary system, and resistance to infection, as well as contributing to the prevention and treatment of several chronic diseases, including hypertension and diabetes [12–20]. The benefits of physical exercise can be attributed to the release of vasoconstrictor substances, increased cardiac rhythm and force, and increased nitric oxide bioavailability [12, 21–25].

Exercise in highly polluted environments reportedly increased the inhalation of air pollutants, including PM_2.5_, due to increased respiratory rate, inappropriate mouth breathing, and reduction of nasal resistance [4, 26–29]. However, the results of studies on the combined health outcomes of long-term exercise and air pollution are inconsistent [16, 17, 25, 30]. Several studies have reported that the beneficial effects of long-term moderate exercise outweigh the adverse effects of air pollutants [13, 15, 19, 31, 32]. In contrast, long-term exercise might also aggravate air pollutant-associated respiratory impairment, including decreased resistance to upper respiratory tract infections, pulmonary dysfunction, and increased prevalence of airway hyper-responsiveness [19, 33, 34].

The inconsistent results of studies on the influence of long-term physical exercise on particle-related cardiopulmonary responses may be due to various confounders, particularly the heterogeneity of the study subjects and their exercise habits [19, 31–33]. Also, most studies were conducted in areas of relatively low pollution, such as North American or European countries [13, 26, 31, 33, 35–37]. The lack of evidence in high-pollution areas has limited discussion on the effects of physical exercise on the cardiopulmonary response to PM_2.5_ exposure [38]. In this study, we assessed the associations between PM_2.5_ exposure and acute cardiopulmonary responses in a panel of healthy young male adults and examined the effects on this associations of long-term exercise habits to determine whether individuals benefit from or are impaired by long-term regular exercise in Beijing, an area with high levels of air pollutants.

## Methods

### Study design and subjects

In the winter of 2014, during the heating season, a period of severe pollution, a panel study was conducted to evaluate acute cardiopulmonary responses to PM_2.5_ exposure in healthy young adults in Beijing, China. Twenty non-smoking male subjects aged 18–26 years were recruited and required to complete a survey of medical history and a baseline questionnaire. No subject reported a history of severe cardiopulmonary illness or regular medication use. The study was approved by the Ethics Committee of Peking University Health Sciences Centre (IRB00001052–14076), and written consent was obtained from all of the subjects.

All subjects completed a total of seven visits spaced 5–7 days apart. During each visit, all subjects underwent a health measurement on the same day. Subjects were asked to refrain from alcohol, caffeine, and exercise for 24 h before measurement. All subjects rested for 20 min and completed a follow-up questionnaire comprising questions on the following: sleep, exercise, and dietary habits, second-hand smoke exposure (> 0.5 h), and clinical respiratory and cardiovascular symptoms within 24 h prior to the visit. Weight and body fat ratio (BFR) were measured for each subject using a body-fat scale (HBF-358-BW; Omron Healthcare, Inc., Dalian, Liaoning, China), and height was measured using a meter stick. Body mass index (BMI) was calculated as the weight divided by the square of the height. Bio-samples were collected from the subjects at a fixed time of day during all seven visits to control for diurnal variation in biological indicators.

### Exercise habits

Information on subjects’ exercise habits was collected in the baseline questionnaire. Subjects were asked to recall their exercise habits in the past year, including exercise frequency and place. Exercise frequency referred to the number of exercise sessions per week. Exercise frequencies of more (less) than once per week were regarded as high (low) exercise frequency. Exercise place referred to the location where subjects habitually undertook physical exercise, including indoor and outdoor locations. Subjects were asked to maintain their exercise habit for 14 days prior to visits and during the entire follow-up period and to report atypical exercise conditions in the follow-up questionnaires.

### Exposure measurement

The concentrations of PM_2.5_ and its constituents were measured continuously using a fixed-site monitor located on the rooftop of a building (~ 18 m in height), which was within 2 km of the subjects’ major residences. The monitoring commenced 14 days prior to the visits and continued throughout the follow-up period. The mass concentration of PM_2.5_ was monitored with 1 min resolution using a tapered element oscillating microbalance sampler (TEOM) (Model 1405; Thermal Electron Corp., Waltham, MA, USA). Particle number concentration in the size range 5.6–560 nm was monitored at 1-min resolution using a fast mobility particle sizer (FMPS) (Model 3091; TSI, Inc., Shoreview, MN, USA) and divided into UFP (5.6–100 nm) and AMP (100–560 nm). The mass concentration of BC was monitored at 5 min resolution using a multi-angle absorption photometer (MAAP) (Model 5012; Thermal Electron Corp., Waltham, MA, USA). Temperature and relative humidity were monitored at hourly resolution using a weather station attached to a four-channel aerosol sampler (TH − 16A; Tianhong, Inc., Wuhan, China). All concentrations were averaged into hourly data for further analysis.

All instruments were maintained and calibrated weekly. The cyclone dust collector and particles cutting head of the FMPS, MAAP, and TEOM, as well as the electric bucket of the FMPS were cleaned softly using dust-free gauze. The zero points of the MAAP and TEOM were calibrated using the self-calibration mode. After connecting the FMPS to the high-efficiency particulate air filter for > 10 min, each channel was calibrated for the zero point. Maintenance took 2 h daily.

### Respiratory and cardiovascular biomarkers

Fractional exhaled nitric oxide (FeNO) samples were collected by an off-line sampling method as described previously [10]. Subjects were asked to inhale through activated carbon to remove ambient NO, and exhale into individual aluminum bags at a flow rate of 150 L/h and positive pressure of 13 cm H_2_O as FeNO samples. The samples were analyzed for NO concentration using an NO/NO_2_/NO_x_ analyzer (Model 42i; Thermo Scientific Corp., Waltham, MA, USA). Exhaled breath condensates (EBC) were collected using a Jaeger EcoScreen collector (Erich Jaeger, Friedberg, Germany). The procedure was described previously [39]. Subjects were asked to inhale and exhale smoothly through the machine using only their mouths. After collection, EBC samples were de-aerated with inert argon gas, aliquoted in labeled cryotubes, and immediately stored at − 80 °C. The levels of interleukin− 2/1β/6(IL-2/1β/6), tumor necrosis factor-α, and interferon-γ in EBC were measured using cytometric bead array and flow cytometry techniques (BD Biosciences, San Diego, CA, USA).

Blood pressure measurements and pulse-wave analysis (PWA) were performed in a quiet, private environment with subjects in the prone position to ensure that the elbow was at the level of the heart. First, medical mercury sphygmomanometers (Jiangsu Yuyue Medical Equipment & Supply Corp., Jiangsu, China) were used to measure peripheral systolic and diastolic blood pressure (SBP and DBP) three times at 2-min intervals, and the averages were used for subsequent analyses. Peripheral pulse pressure was calculated as the difference between SBP and DBP (pulse pressure = SBP − DBP). Next, applanation tonometry using a high-fidelity micromanometer (SphygmoCor; AtCor Medical, Sydney, Australia) was used to record radial arterial pressure waveforms at the wrist of the dominant arm non-invasively. The homogeneity of the observed waveforms was assessed using the built-in quality score. The peripheral waveforms were transformed into corresponding central aortic waveforms via a mathematical function [40]. PWA measurements, including aortic augmentation pressure, augmentation index, and heart ejection duration, were determined using the 15-s sequential aortic pressure waveforms and were considered indicators of endothelial function and blood supply capacity [41, 42]. Specifically, the aortic augmentation pressure was defined as the average difference between the second and first systolic peaks of the 15-s sequential aortic pressure waveforms, and the aortic pressure index was defined as the ratio of the aortic augmentation pressure to the peripheral pulse pressure [41, 43].

### Statistical analysis

Linear mixed-effects (LMEs) models were used to estimate the associations between acute cardiopulmonary responses and exposure to PM_2.5_ and its constituents. The fixed and random effects of the LME models were estimated by the restricted maximum-likelihood method. Random subject-specific intercepts were included in the models to control for within-subject variation among the seven repeated measurements, but the random slopes were not considered. The dependent variables were the levels of the biomarkers, which were logarithmically transformed because of their non-normal distribution. The independent variables were the moving average (MA) concentrations of PM_2.5_, including the mass concentrations of PM_2.5_ and BC and the number concentrations of UFP and AMP, prior to each visit. All of the LME models were adjusted for ambient temperature and relative humidity to account for their potential impact on the levels of the biomarkers.

In stratified analyses, the single-pollutant LME models included BMI or BFR or exercise frequency or exercise place as a first-order interaction term to estimate the exposure–response associations modified by exercise habits, which was the primary aim of this study. For instance, in the formula used to assess the effects of exercise frequency on the associations: *biomarker* = β_1*_exposure + β_2_*exercise frequency + β_3_*exposure × exercise frequency, the regression coefficients in the additive LME model for subjects with low and high exercise frequencies were β_1_ and (β_1_ + β_3_), respectively. In a sensitivity analysis, a single-pollutant LME model was used to evaluate whether the exposure–response associations changed after further adjustment for age, breakfast, sleep quality, second-hand smoke exposure, and the daily sleeping, working, and outdoor times. Two-pollutant LME models were used to examine the consistency of the estimated results of the single-pollutant LME models by further adjustment for all combinations of pollutant metrics.

The final results (β-coefficient) of the LME models are reported as the estimated percentage changes in the dependent variables for each interquartile range (IQR) increase in pollutant concentrations. Results were calculated as β = [(exp^(β^′^ × IQR)) − 1] × 100%, where β^′^ is the estimated effect of the corresponding model. The 95% confidence intervals (CIs) were transformed using the same formula as for β. Statistical significance was considered present at *p* < 0.05. All data analyses were conducted using R statistical software (ver. 3.1; R Development Core Team, Vienna, Austria).

## Results

### General data: Descriptive statistics

In total, 20 subjects completed seven visits, but 13 of the 140 records labelled with acute respiratory symptoms or medication use were intentionally deleted. Before visits. The general characteristics of the subjects are shown in Table 1. The ranges of daily sleeping, working, and outdoor times of the subjects were small, which suggested that these healthy young male non-smokers had similar living conditions. The proportions of the visits at which the subjects reported having had breakfast, poor sleep quality, and second-hand smoke exposure before biomarker collection were 24, 39, and 7%, respectively. Among the subjects, 50 and 45% exercised more than once per week and usually exercised outdoors, respectively. Six subjects exercised more than once per week and also preferred outdoor exercise. The median BMI and BFR values were 23.0 kg/m^2^ and 18.0%, respectively. The averaged levels of FeNO, SBP, and DBP, and the ejection duration, aortic augmentation pressure, and aortic pressure indices were 17.1 ± 10.4 ppb, 123 ± 10 mmHg, 72 ± 7 mmHg, 37 ± 4%, − 1 ± 2 mmHg, and − 2 ± 8%, respectively. The averaged IL-2 level was 8.0 ± 0.8 pg/mL, and the levels of other cytokines in EBC were below the limits of detection.

**Table 1:** Characteristics of the subjects and baseline biomarkers measurements

Characteristics/biomarkers	*n*	Mean/% (SD)	Median (Range)
Age (year)	20	23 (2)	23 (18–26)
Daily Sleeping Time (hr)	20	7 (1)	7 (3–10)
Daily Working Time (hr)	20	5 (2)	5 (3–8)
Daily Outdoor Time (hr)	20	3 (2)	3 (1–6)
Breakfast before Visit			
Yes	31	24%	
No	96	76%	
Poor Sleep Quality before Visit			
Yes	49	39%	
No	78	61%	
Second-Hand Smoke Exposure ^*a*^ (Past 24-h)			
Yes	9	7%	
No	118	93%	
Weekly Aerobic Exercise ≥2/week (Past 3-years)			
Yes	10	50%	
No	10	50%	
Outdoor Exercise			
Yes	9	45%	
No	11	55%	
Body Mass Index (kg/m^2^)	20	22.4 (2.8)	23.0 (15.6–27.8)
Body Fat Rate (%)	20	18.5 (4.1)	18.0 (8.6–26.5)
FeNO (ppb)	127	17.1 (10.4)	14.0 (2.3–48.3)
IL-2 (pg/mL)	115	8.0 (0.8)	8.1 (7.2–10.3)
SBP(mmHg)	127	123 (10)	122 (104–132)
DBP(mmHg)	127	72 (7)	72 (58–80)
Ejection duration (%)	127	37 (4)	37 (27–47)
Aortic augmentation pressure (mmHg)	127	−1 (2)	0 (−7–4)
Aortic pressure index (%)	127	-2 (8)	-1 (−19–13)

Abbreviations: FeNO, fractional exhaled nitric oxide; IL-2, interleukin-2; SBP and DBP: peripheral systolic and diastolic blood pressure

^*a*^ it only included exposures which lasted more than 0.5 hr

The distribution of air-pollutant exposure during the follow-up period is shown in Table 2. Missing data included PM_2.5_ measurement at 83 h, BC measurement at 236 h, and UFPs and AMP measurements at 191 h due to temporary power outages or extreme weather. The mass concentrations of PM_2.5_ and BC were 65.2 ± 69.6 and 5.3 ± 4.2 μg/m^3^, respectively. The number concentrations of UFP and AMP were 16.2 ± 7.3 and 4.6 ± 3.8 10^3^/cm^3^, respectively. Compared with most former studies of the combined effects of ambient air pollution exposure and physical exercise [26, 31, 35–37], the levels of PM_2.5_ and its constituents were higher, and the ranges were wider in our study. For instance, in a study conducted in Europe, the annual average concentration of PM_2.5_ was 14.8 ± 1.8 μg/m^3^ [31]. In another European study, the median (IQR) of BC exposure during a 24-h period was 1.4 (1.1–1.8) μg/m^3^ [35]. Weichenthal et al. performed a cross-over study in Canada, which showed that the weekly average levels of PM_2.5_, BC, and UFP around a traffic route were 14.2 ± 1.3 μg/m^3^, 1.7 ± 1.4 μg/m^3^, and 16.8 ± 10.3 10^3^/cm^3^, respectively [36].

**Table 2:** Distributions of air pollutants and meteorological parameters (hourly average) during the follow-up period

Variables	*n*	Mean (SD)	IQR
PM_2.5_ (μg/m^3^)	1261	65.2(69.6)	5.8–106.2
BC (μg /m^3^)	1108	5.3(4.2)	1.0–8.9
UFP (10^3^/cm^3^)	1153	16.2(7.3)	11.4–20.8
AMP (10^3^/cm^3^)	1153	4.6(3.8)	0.7–7.6
Temperature (°C)	1344	1.3(3.3)	−1.2-3.5
Relative Humidity (%)	1344	24.8(12.7)	1.5–31.5

Abbreviation: PM_2.5_, fine particles; BC, black carbon; UFP, ultrafine particles; AMP, particles in accumulation mode

As shown in Additional file 1: Table S1 and S2, the concentrations of PM_2.5_, BC, and AMP were highly correlated (Pearson correlation coefficient ≥ 0.8, *p* ≤ 0.001). The Pearson correlation coefficients between the number concentration of UFP and other pollutants were relatively low, suggesting different sources of UFP. With the exception of the correlation between aortic augmentation pressure and the aortic pressure index (Pearson correlation coefficient = 0.975), the biomarker levels were not highly correlated.

### Results of LME models for all subjects

The results of single-pollutant LME models for all subjects are shown in Additional file 1: Figure S1–S7. Increased FeNO concentrations were significantly and consecutively associated with 2–6-h MA exposure to PM_2.5_ and its constituents (β = 16–40%). Increased SBP was associated with 5- and 7-d MA exposure to AMP (β = 1–2%). Increased ejection duration was significantly associated with 2-h and 9-d MA exposure to PM_2.5_ and its constituents (β = 1–2%). Increased aortic augmentation pressure was significantly and consecutively associated with 7–9-d MA exposure to PM_2.5_ and BC (β = 5–10%), and 7–13-d MA exposure to UFP and AMP (β = 5–13%). An increased aortic pressure index was significantly and consecutively associated with 5–9-d MA exposure to PM_2.5_ and BC (β = 5–26%) and 9–13-d MA exposure to UFP and AMP (β = 7–13%). The levels of IL-2 and DBP were not associated with PM_2.5_ exposure.

The results of the two-pollutant LME models are shown in Additional file 1: Figure S8. The associations between the levels of the biomarkers and UFP exposure remained robust and significant after adjustment for the levels of other pollutants. The associations of the levels of FeNO with ejection duration and BC exposure were also relatively robust. In addition, according to the sensitivity analysis (Additional file 1: Table S3), taking the effect of UFP exposure as an example, the results of LME models were not affected by age, breakfast, sleep quality, second-hand smoke exposure, or the daily sleeping, working, and outdoor times.

### Stratified analyses of LME models

**Fig. 1:**
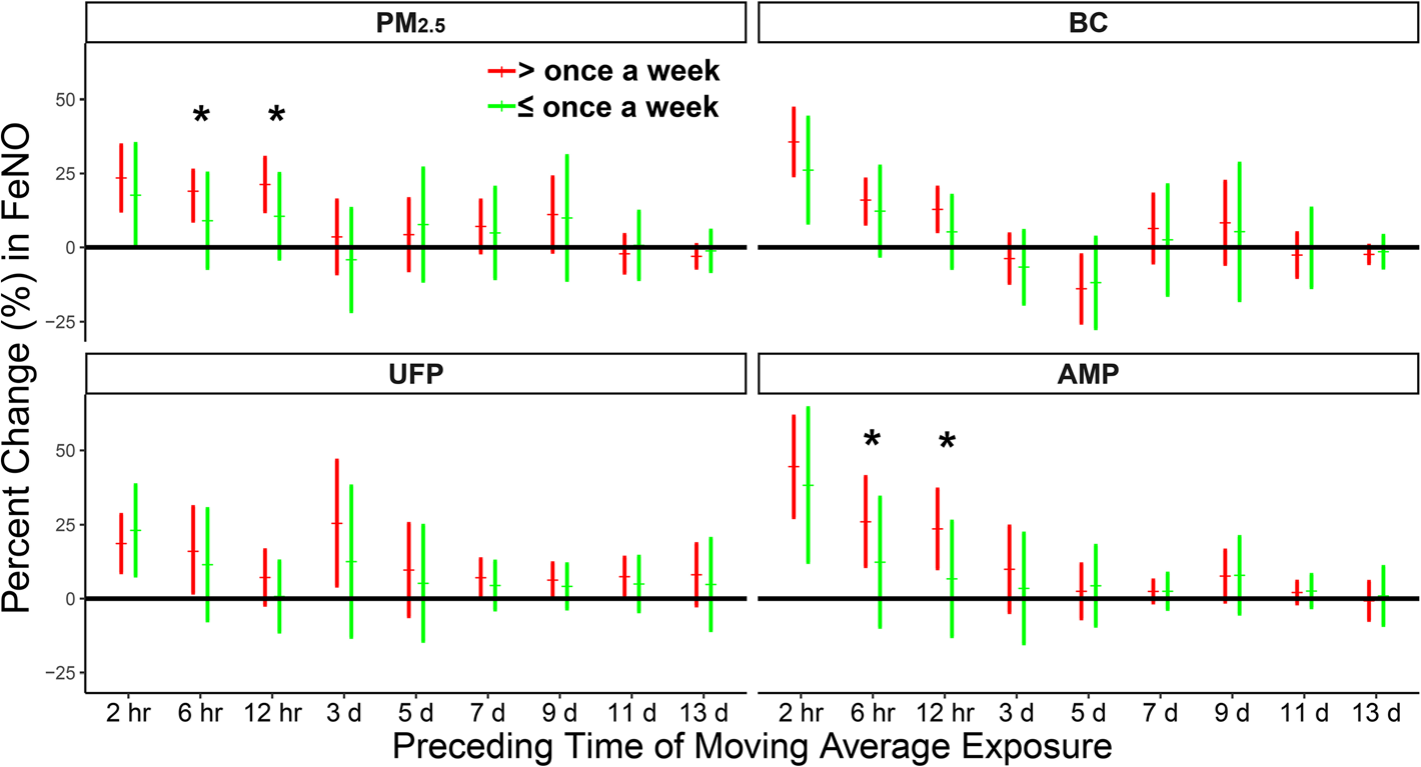
Changes in FeNO associated with IQR increases in concentrations of PM_2.5_ and its constituents stratified by exercise frequency. The models are adjusted for ambient temperature and relative humidity. Red and green error bars show estimated changes in subjects who exercise more and less than once per week, respectively. ^★^ Significant (*p* < 0.05) differences between estimates

The association between FeNO elevation and particle exposure was analyzed stratified by habitual exercise frequency (Fig. 1). IQR increases in 6-h MA exposure to PM_2.5_ and AMP were associated with 19% (CI = 9–29%) and 26% (CI = 11–41%) increases in the FeNO concentration, respectively, in subjects with high exercise frequency; these increases were significantly greater than those in subjects with low exercise frequency. IQR increases in 12-h MA exposure to PM_2.5_ and AMP were associated with 21% (CI = 12–30%) and 24% (CI = 10–37%) increases in FeNO concentration, respectively, in subjects with high exercise frequency; these increases were significantly greater than those in subjects with low exercise frequency. The changes in FeNO concentration associated with exposure to BC and UFP did not differ between subjects with high and those with low exercise frequencies.

**Fig. 2:**
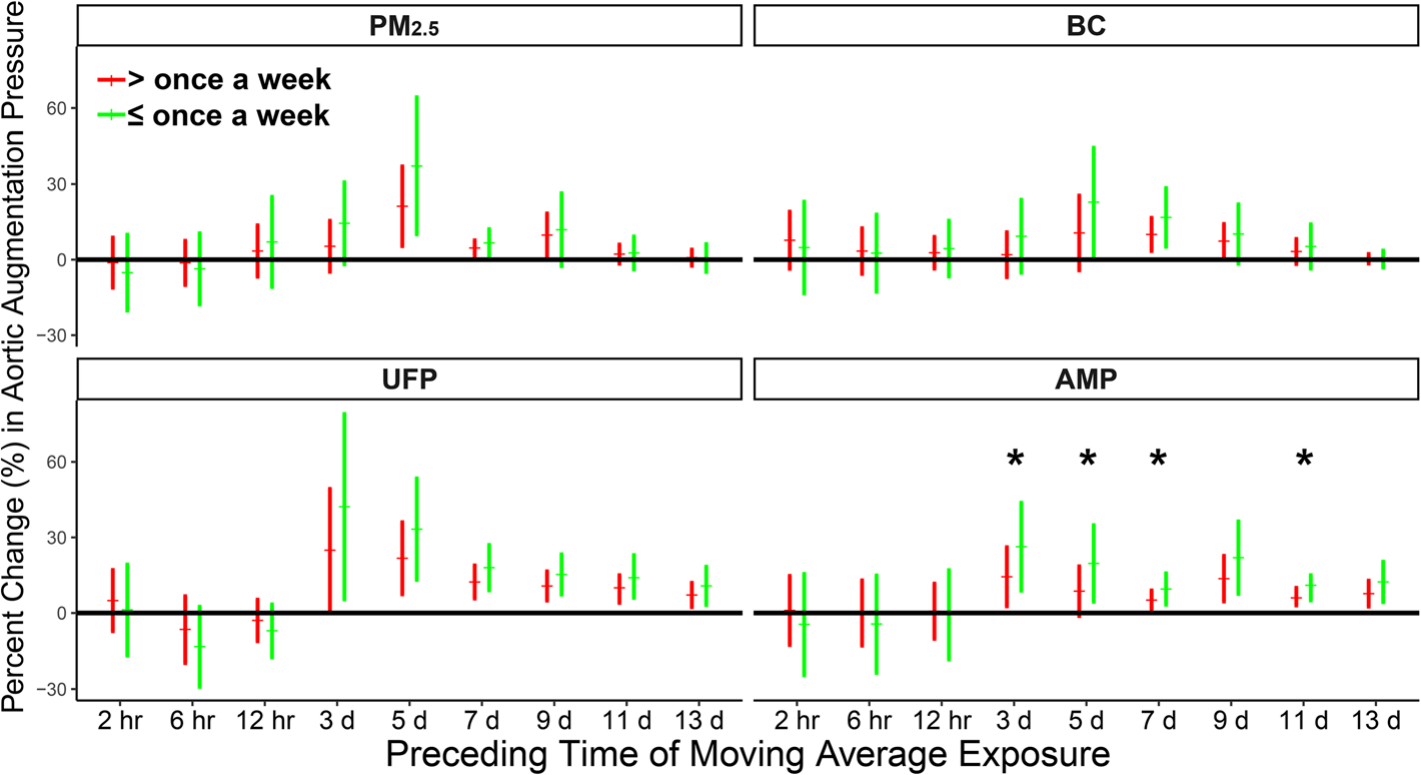
Changes in aortic augmentation pressure associated with IQR increases in concentrations of PM_2.5_ and its constituents stratified by exercise frequency. The models are adjusted for ambient temperature and relative humidity. Red and green error bars indicate estimated changes in subjects who exercise more and less than once per week, respectively. ^★^ Significant (*p* < 0.05) differences between estimates

The association between aortic augmentation pressure elevation and particle exposure was analyzed stratified by habitual exercise frequency (Fig. 2). IQR increases in 3-, 5-, 7-, and 11-d MA AMP exposure were associated with 26% (CI = 9–44%), 20% (CI = 4–35%), 10% (CI = 3–16%), and 11% (CI = 5–15%), respectively, increases in aortic augmentation pressure in subjects with low exercise frequency; these increases were significantly greater than those in subjects with high exercise frequency. The aortic augmentation pressure elevations associated with 5-d BC, 5–11-d UFP, and 9- and 13-d AMP MA exposure were marginally significantly higher in subjects with low exercise frequency.

**Fig. 3:**
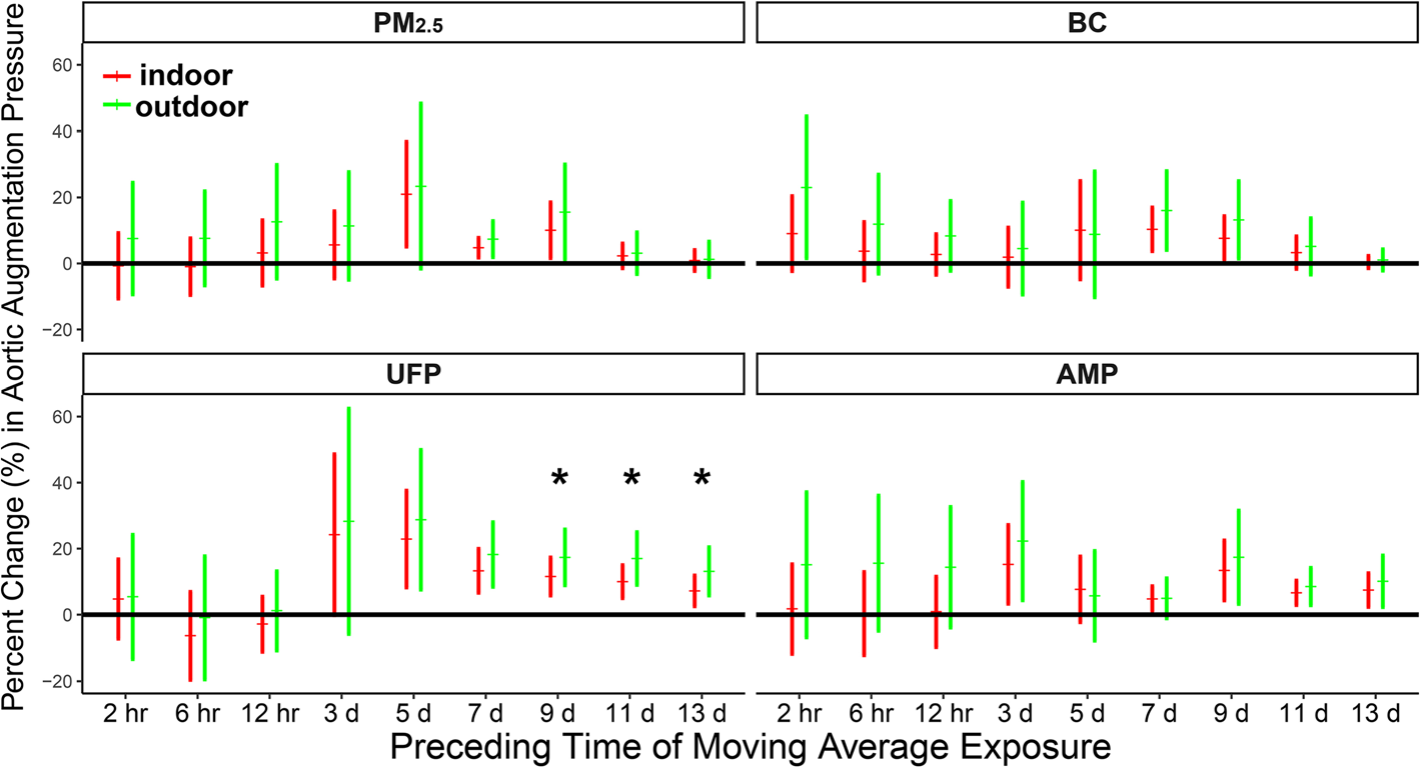
Changes in aortic augmentation pressure associated with IQR increases in concentrations of PM_2.5_ and its constituents stratified by exercise place. The models are adjusted for ambient temperature and relative humidity. Red and green error bars show estimated changes in subjects who prefer indoor and outdoor exercise, respectively. ^★^ Significant (*p* < 0.05) differences between estimates

The association between aortic augmentation pressure elevation and particle exposure was analyzed stratified by exercise place (Fig. 3). IQR increases in the 9-, 11-, and 13-d MA UFP exposure were associated with 17% (CI = 9–26%), 17% (CI = 9–25%), and 13% (CI = 6–30%), respectively, increases in aortic augmentation pressure in subjects who preferred outdoor exercise; these increases were significantly greater than those in subjects who preferred indoor exercise. The changes in aortic augmentation pressure associated with PM_2.5_, BC, and AMP exposure were not analyzed stratified by exercise place. In addition, no stratification in the changes in IL-2 level, SBP, DBP, ejection duration, and aortic pressure index associated with particle exposure was observed in LME The associations between the levels of biomarkers and particle exposure were not modified by BMI or BFR (Additional file 1: Figure S9–S32).

### Levels of FeNO and aortic augmentation pressure

In several time windows, e.g., 6 h and 11 d, LME models showed that the associations of FeNO level or aortic augmentation pressure with particle exposure differed according to exercise frequency and place. Comparisons of the responses of different subgroups to high and low particle concentrations helped to explain the results of the LME models.

**Fig. 4:**
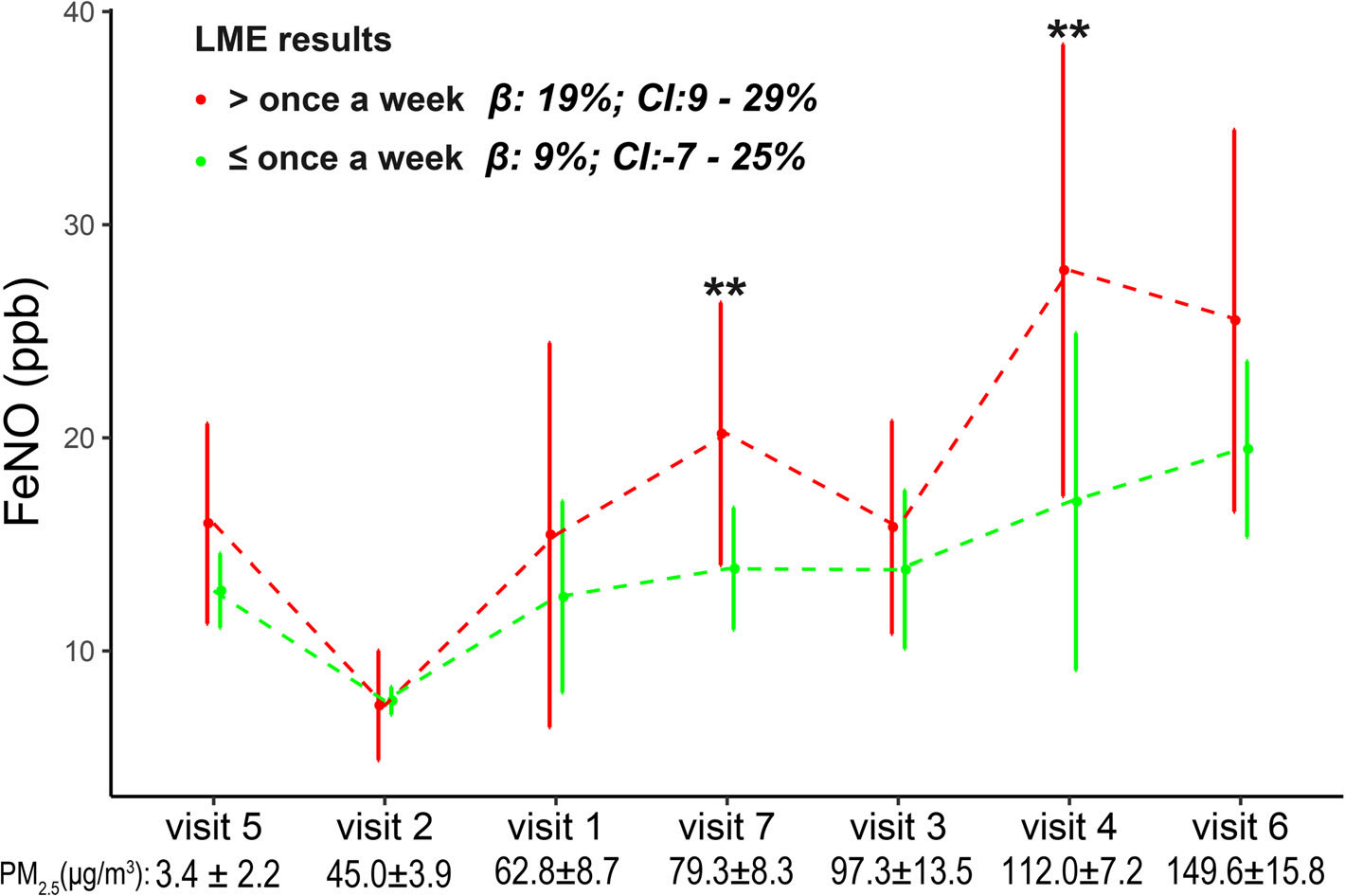
Level of FeNO (mean ± SD) at seven visits according to exercise frequency. The visits are ordered by the prior 6-h MA PM_2.5_ exposure (bottom). β, estimated change in the FeNO level per IQR increase in PM_2.5_ exposure in the LME models. The LME models are adjusted for ambient temperature and relative humidity. ** Significant difference in FeNO between subjects who exercise more and less than once per week by unpaired *t*-test

The variation in FeNO concentration across seven visits and the corresponding 6-h MA PM_2.5_ concentration prior to each visit are shown in Fig. 4. The concentrations of FeNO in subjects with low and high exercise frequencies were similar following exposure to a low level of PM_2.5_ but differed following exposure to a high level of PM_2.5_. For instance, at visit 2 (PM_2.5_, 45.0 ± 3.9 μg/m^3^), the concentration of FeNO was 7.4 ± 2.6 and 7.7 ± 0.7 ppb in subjects with high and low exercise frequencies, respectively; at visit 4 (PM_2.5_, 112.0 ± 7.2 μg/m^3^), the concentration of FeNO in subjects with high exercise frequency was 27.9 ± 10.7 ppb, significantly higher than that in subjects with low exercise frequency (17.0 ± 7.9 ppb).

**Fig. 5:**
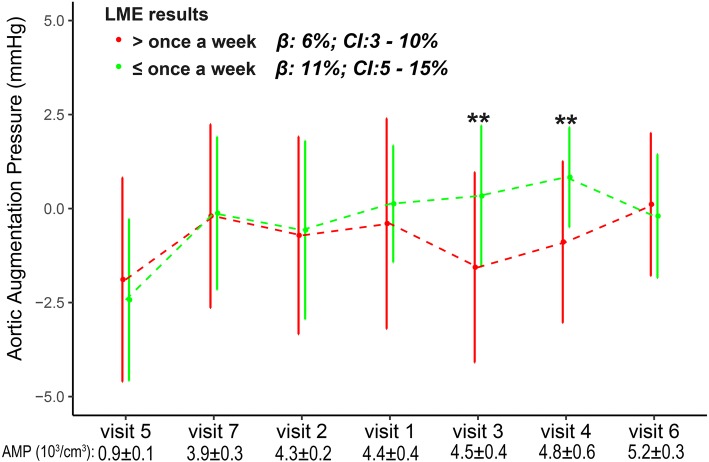
Aortic augmentation pressure (mean ± SD) at seven visits according to exercise frequency. The visits are ordered by prior 11-d MA AMP exposure (bottom). Β, estimated change in aortic augmentation pressure per IQR increase in AMP exposure in the LME models. All LME models were adjusted for ambient temperature and relative humidity. ** Significant difference in aortic augmentation pressure between subjects who exercise more and less than once per week by unpaired *t*-test

**Fig. 6:**
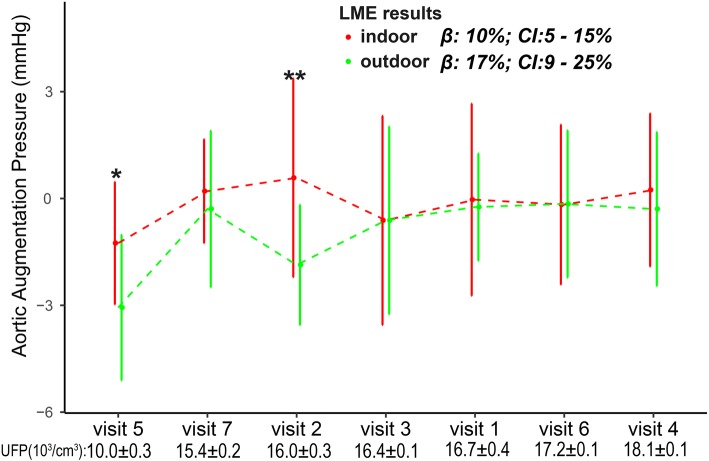
Aortic augmentation pressure (mean ± SD) at seven visits in subjects who preferred indoor (red) and outdoor (green) exercise. The visits are ordered by the 11-d MA UFP exposure before visits, which are listed at the bottom. Β, estimated change in aortic augmentation pressure per IQR increase in UFP exposure in the LME models. LME models were adjusted for ambient temperature and relative humidity. ** Significant difference in aortic augmentation pressure between indoor and outdoor exercise by unpaired *t*-test

The variation in aortic augmentation pressure across seven visits and the corresponding 11-d MA AMP and UFP concentrations prior to each visit are shown in Figs. 5 and 6. The differences in aortic augmentation pressure between subjects with high and those with low exercise frequencies were greater following high-level compared to low-level PM_2.5_ exposure. For instance, at visit 5 (AMP, 0.9 ± 0.1 10^3^/cm^3^), the aortic augmentation pressure was − 2 ± 3 and − 2 ± 2 mmHg in subjects with high and low exercise frequency, respectively; at visit 3 (AMP, 4.5 ± 0.4 10^3^/cm^3^), the aortic augmentation pressure in subjects with high exercise frequency was − 2 ± 3 mmHg, significantly lower than that in subjects with low exercise frequency (0 ± 2 mmHg). The difference in aortic augmentation pressure between subjects who preferred indoor and outdoor exercise was smaller at visits following high-level than at those following low-level UFP exposure (Fig. 5). For instance, at visit 5 (UFP, 10.0 ± 0.3 10^3^/cm^3^), the aortic augmentation pressure in subjects who preferred indoor exercise was − 1 ± 2 mmHg, higher than that in subjects who preferred outdoor exercise (− 3 ± 2 mmHg). At visit 6 (UFP, 17.2 ± 0.1 10^3^/cm^3^), the aortic augmentation pressure was 0 ± 2 and 0 ± 2 mmHg in subjects who preferred indoor and outdoor exercise, respectively.

## Discussion

The results of this study were consistent with prior reports of positive associations between PM_2.5_ exposure and increased respiratory inflammation and arterial stiffness in healthy young adults (Additional file 1: Table S4) [44–52]. Changes in FeNO concentration, aortic augmentation pressure, and aortic pressure index associated with PM_2.5_ exposure in this study were modified by exercise habits, suggesting a combined effect of long-term habitual exercise and PM_2.5_ exposure. However, some studies observed no particle-mediated acute increases in FeNO, ejection duration, aortic augmentation pressure, or aortic pressure index in healthy young adults. The inconsistency between those results and ours might reflect differences in the subjects recruited and the time windows chosen, as well as the relatively lower levels of particle exposure in previous studies [52–60].

Consistent with previous studies [10, 11, 39], the concentration of FeNO, a biomarker of eosinophilic airway inflammation, was positively associated with air-pollution exposure, and PM_2.5_-mediated changes in the FeNO concentration were greater in subjects with high exercise frequency. These results may explain why individuals who engage in high-strength exercise may be at higher risk of particle-mediated respiratory symptoms. Consistent with our results, in a study on chronic respiratory responses to PM_2.5_ inhalation during exercise [61], competitive athletes were found to be susceptible to pulmonary inflammation and had decreased lung function and an increased risk of asthma. The control group, comprising individuals who engaged in exercise less frequently, was not susceptible to these effects of PM_2.5_ inhalation. Bougault et al. reported that the number of training hours per week was correlated with the percentage of sputum neutrophils in athletes who trained in cold air [33]. Damage to airway defenses may explain the enhanced risk to respiratory health caused by habitual high-frequency exercise [12, 21] Nasal structures, e.g., epithelial cilia, contribute 50–60% of total respiratory resistance, the first barrier to particle ingress [62]. Muns et al. demonstrated that long-distance runners exhibited decreased nasal mucociliary clearance and ciliary movement [63]. In a study on particle clearance in the respiratory system, the nasal resistance of runners both within and outside a city was significantly reduced after exercise, which increased their susceptibility to PM_2.5_ exposure [27].

In terms of cardiovascular responses, increased aortic augmentation pressure and aortic pressure index indicate higher pulse-wave velocity and earlier return of the reflected wave, which suggest higher arterial stiffness following PM_2.5_ exposure [41, 43]. The positive associations between exposure to PM_2.5_ and arterial stiffness were weaker in subjects with high exercise frequency, suggesting that long-term habitual exercise is associated with improved cardiovascular fitness and an increase in cardiovascular resistance to air pollutants [12, 15, 16, 64].

Low cardiopulmonary fitness is a key predictor of cardiovascular mortality in healthy subjects and patients with coronary heart disease [65, 66]. Consistent with our results, in a study of the excess risk of mortality per 10 μg/m^3^ increase in air-pollutant levels in Hong Kong, Wong et al. reported that exercise at a moderate frequency (≥1 and < 4/week) had a greater protective effect against cardiopulmonary death attributable to particulate matter and ozone than a low or excessive exercise frequency (< 1 or ≥ 4/week) [19]. In a cohort study of air pollution and lung and heart disease, Endes et al. observed that increased brachial-ankle pulse-wave velocity, a biomarker of arterial stiffness, was significantly associated with self-reported PM_2.5_ and PM_10_ exposure in physically inactive, but not in physically active, elderly subjects [31]. In addition, as a surrogate biomarker of pre-clinical atherosclerosis, arterial wall thickness decreased following long-term exercise training, which contributed to cardioprotection, but increased after prolonged and repetitive exposure to cardiovascular risk factors, including air pollution [18].

Outdoor physical exercise, including jogging, walking, running, social dancing, and marathon running, remain popular in China despite the severe air pollution [67]. In this study, aortic augmentation pressure following low-level ambient UFP exposure in subjects who preferred outdoor exercise was lower than that in subjects who preferred indoor exercise, but the difference disappeared with high-level UFP exposure. Several studies in areas with low concentrations of ambient PM_2.5_ (< 30 μg/m^3^) reported higher levels of personal and residential indoor than outdoor PM_2.5_ exposure, possibly due to human activities [68–71]. In highly polluted areas, for instance, in China, the correlation between outdoor and indoor PM_2.5_ concentrations was stronger at higher ambient pollution levels, suggesting an increased contribution of outdoor pollution to indoor PM_2.5_ exposure [72, 73]. The difference between the indoor and outdoor PM_2.5_ concentration decreased with increasing exposure to ambient PM_2.5_, which may explain why a difference in aortic augmentation pressure between subjects who preferred indoor and outdoor exercise was only observed following exposure to low-level ambient PM_2.5_.

A panel of subjects completed seven visits under different air-pollution conditions, so all subjects acted as their own controls, which reduced the error associated with intra-individual differences. In addition, repeated measurements of each subject enhanced the power of the LME models [44, 74, 75]. The homogeneous characteristics of the subjects reduced the number and magnitude of fluctuations in the confounders considered in evaluating the combined effects of habitual exercise and PM_2.5_ exposure in young healthy males [53, 54]. This study had several limitations. First, the small sample size (*n* = 20 × 7) limited the statistical power to evaluate the responses associated with exposure to PM_2.5_. Second, the levels of pollutants were monitored at a fixed monitoring station instead of at a personal level. Although all subjects lived within 2 km of the monitoring station and had similar time–activity modes, exposure misclassification may have occurred. Because the monitoring station was located near a main road, the error in BC measurements was considerable. Third, the subjects had relatively high levels of education, similar living habits, and good healthcare, so our findings cannot be generalized to the general population. Fourth, FMPS only measured the number concentration of particles in the size range 5.6–560 nm, which prevented evaluation of the effects of larger particles. Fifth, due to the similar time–activity modes of the subjects, only exercise frequency and place were recorded in the baseline questionnaire to describe exercise habits. These reflected only some aspects of exercise habits; other important information, such as whether subjects preferred running or swimming, was missed.

## Conclusions

Long-term habitual exercise in severely polluted areas may strengthen the resistance of the cardiovascular system to exposure to PM_2.5_ but increase the risk of pollutant-related airway inflammation.

## Additional file

Additional file 1 **Table S1.** Pearson correlation coefficients among air pollutants. **Table S2.** Pearson correlation coefficients among health outcomes. **Table S3.** Percent changes (β) in biomarkers per IQR increases in UFP, with further adjustment for multiple confounders. **Table S4.** Studies concerning the effects of particles exposure on FeNO, aortic augmentation pressure, and aortic pressure index in healthy young adults. **Figure S1-S7.** Percent changes in FeNO, IL-2, SBP, DBP, ejection duration, aortic augmentation pressure and aortic pressure index per IQR increases in particles exposure in single pollutant models, respectively. **Figure S8.** Percent changes in A) aortic pressure index, B) aortic augmentation pressure, C) ejection duration, D) FeNO per IQR increases in particles exposure in two-pollutant models. **Figure S9-S11.** Percent changes in FeNO with the IQR increases in concentrations of PM_2.5_ and its constituents stratified by exercise place, BMI, and BFR. **Fig. S12-S15.** Percent changes in IL-2 with the IQR increases in concentrations of PM_2.5_ and its constituents stratified by exercise frequency, exercise place, BMI, and BFR. **Figure S16-S19.** Percent changes in SBP with the IQR increases in concentrations of PM_2.5_ and its constituents stratified by exercise frequency, exercise place, BMI, and BFR. **Figure S20-S23.** Percent changes in DBP with the IQR increases in concentrations of PM_2.5_ and its constituents stratified by exercise frequency, exercise place, BMI, and BFR. **Figure S24-S27.** Percent changes in ejection duration with the IQR increases in concentrations of PM_2.5_ and its constituents stratified by exercise frequency, exercise place, BMI, and BFR. **Figure S28-S29.** percent changes in aortic augmentation pressure with the IQR increases in concentrations of PM_2.5_ and its constituents stratified by BMI and BFR. **Figure S30-S33.** percent changes in aortic pressure index with the IQR increases in concentrations of PM_2.5_ and its constituents stratified by exercise frequency, exercise place, BMI, and BFR. (DOCX 3760 kb)

## Abbreviations

AMP: accumulation mode particles
BC: black carbon
BFR: body fat ratio
BMI: body mass index
CI: confidence interval
EBC: exhaled breath condensates
FeNO: fractional exhaled nitric oxide
IL-2/1β/6: interleukin-2/1β/6
IQR: interquartile range
LMA: linear mixed-effects model
MA: moving average
PM_2.5_: particulate matter with an aerodynamic diameter ≤ 2.5 μm
ppb: parts per billion
PWA: pulse wave analysis
SBP and DBP: peripheral systolic and diastolic blood pressure
SD: standard deviation
UFP: ultrafine particles
