# Supplementary material for: Responses of healthy young males to fine-particle exposure are modified by exercise habits: a panel study
Source: Environ Health. 2018 Dec 13;17:88. doi: 10.1186/s12940-018-0437-3 (PMC6293663; doi:10.1186/s12940-018-0437-3)
Supplement: Supplementary file 1 — Table S1. Pearson correlation coefficients among air pollutants. Table S2. Pearson correlation coefficients among health outcomes. Table S3. Percent changes (β) in biomarkers per IQR increases in UFP, with further adjustment for multiple confounders. Table S4. Studies concerning the effects of particles exposure on FeNO, aortic augmentation pressure, and aortic pressure index in healthy young adults. Figure S1-S7. Percent changes in FeNO, IL-2, SBP, DBP, ejection duration, aortic augmentation pressure and aortic pressure index per IQR increases in particles exposure in single pollutant models, respectively. Figure S8. Percent changes in A) aortic pressure index, B) aortic augmentation pressure, C) ejection duration, D) FeNO per IQR increases in particles exposure in two-pollutant models. Figure S9-S11. Percent changes in FeNO with the IQR increases in concentrations of PM2.5 and its constituents stratified by exercise place, BMI, and BFR. Fig. S12-S15. Percent changes in IL-2 with the IQR increases in concentrations of PM2.5 and its constituents stratified by exercise frequency, exercise place, BMI, and BFR. Figure S16-S19. Percent changes in SBP with the IQR increases in concentrations of PM2.5 and its constituents stratified by exercise frequency, exercise place, BMI, and BFR. Figure S20-S23. Percent changes in DBP with the IQR increases in concentrations of PM2.5 and its constituents stratified by exercise frequency, exercise place, BMI, and BFR. Figure S24-S27. Percent changes in ejection duration with the IQR increases in concentrations of PM2.5 and its constituents stratified by exercise frequency, exercise place, BMI, and BFR. Figure S28-S29. percent changes in aortic augmentation pressure with the IQR increases in concentrations of PM2.5 and its constituents stratified by BMI and BFR. Figure S30-S33. percent changes in aortic pressure index with the IQR increases in concentrations of PM2.5 and its constituents stratified by exercise frequency, exercise place [file 12940_2018_437_MOESM1_ESM.docx]

**Additional file 1**

**Table S1 pearson correlation coefficients among air pollutants**

|  | PM_2.5_ | BC | UFP | AMP | Temperature | RH |
| --- | --- | --- | --- | --- | --- | --- |
| PM_2.5_ | 1.000 | 0.868 | 0.257 | 0.853 | -0.100 | 0.661 |
| BC |  | 1.000 | 0.390 | 0.871 | -0.137 | 0.641 |
| UFP |  |  | 1.000 | 0.407 | 0.083 | 0.149 |
| AMP |  |  |  | 1.000 | -0.141 | 0.593 |
| Temperature |  |  |  |  | 1.000 | -0.327 |
| RH |  |  |  |  |  | 1.000 |

Abbreviations: BC, black carbon; UFP, ultrafine particles; AMP, accumulation mode particles; RH, relative humidity.

**Table S2 pearson correlation coefficients among health outcomes.**

|  | FeNO | IL-2 | SBP | DBP | Ejection  duration | Aortic  augmentation  pressure | Aortic  pressure  index |
| --- | --- | --- | --- | --- | --- | --- | --- |
| FeNO | 1.000 | 0.079 | -0.058 | -0.101 | 0.102 | 0.115 | 0.127 |
| IL-2 |  | 1.000 | 0.047 | 0.065 | -0.036 | -0.121 | -0.147 |
| SBP |  |  | 1.000 | 0.414 | -0.005 | -0.147 | -0.123 |
| DBP |  |  |  | 1.000 | -0.375 | 0.178 | 0.123 |
| Ejection  duration |  |  |  |  | 1.000 | -0.037 | 0.024 |
| Aortic  Augmentation  pressure |  |  |  |  |  | 1.000 | 0.975 |
| Aortic  pressure  index |  |  |  |  |  |  | 1.000 |

Abbreviations: FeNO, fractional exhaled nitric oxide; IL-2, interleukin-2; SBP and DBP: peripheral systolic and diastolic blood pressure.

**Table S3. percent changes** (β) **in biomarkers per IQR increases in UFP, with further adjustment for multiple confounders.**

| Biomarkers | FeNO | | Ejection duration | | Aortic  augmentation  pressure | | Aortic  pressure  index | |
| --- | --- | --- | --- | --- | --- | --- | --- | --- |
| Exposure Time | 2 hr MA | | 2 hr MA | | 9 d MA | | 9 d MA | |
|  | β | 95% CI | β | 95% CI | β | 95% CI | β | 95% CI |
| Model 1 *^a^* | 25% | 11-38% | 2% | 1-3% | 11% | 5-16% | 12% | 5-19% |
| Model 2 | 24% | 10-38% | 1% | 0-3% | 11% | 5-16% | 12% | 5-19% |
| Model 3 | 25% | 11-39% | 1% | 0-3% | 10% | 5-16% | 12% | 5-19% |
| Model 4 | 25% | 11-39% | 2% | 0-3% | 10% | 5-16% | 12% | 5-19% |
| Model 5 | 24% | 10-38% | 1% | 0-3% | 10% | 5-15% | 11% | 4-18% |
| Model 6 | 25% | 11-39% | 1% | 0-3% | 11% | 5-16% | 12% | 5-19% |
| Model 7 | 23% | 10-37% | 2% | 0-3% | 11% | 5-16% | 12% | 5-19% |
| Model 8 | 25% | 11-39% | 2% | 0-3% | 11% | 5-16% | 12% | 5-19% |

Abbreviation: FeNO, fractional exhaled nitric oxide; UFP, ultrafine particles; IQR, interquartile range; CI, confidence interval, MA, moving average.

*^a^* Model 1 denotes the single-pollutant model adjusted for ambient temperature and relative humidity. Model 2-8 denote the model 1 with further adjustment for age, breakfast, sleep quality, second-hand smoke exposure, and daily sleeping, working, outdoor time, respectively

**Table S4 studies concerning the effects of particles exposure on FeNO, ejection duration, aortic augmentation pressure, and aortic pressure index in healthy young adults**

| Study | Pollutants | Biomarkers | Effects |
| --- | --- | --- | --- |
| Golan *et al.* 2018 | 2 hr average PM_2.5_ (≤ 18.4±9.3 μg/m^3^)  2 hr average PNC (≤ 35.2±14.4 10^3^/cm^3^) | FeNO  FeNO | No effect  No effect |
| Shi *et al.* 2016 | 24 hr average PM_2.5_ (66.8±40.1 μg/m^3^) | FeNO | 8.9% increase |
| Wauters *et al.* 2015 | 2 hr average PM_2.5_ (diesel exhaust: 304±1 μg/m^3^) | FeNO | No effect |
| Zhang *et al.* 2013 | Daily average PM_2.5_ (≤ 98.9±14.7 μg/m^3^)  Daily average PNC (≤ 19.5±13.7 10^3^/cm^3^) | FeNO  FeNO | 40.7% increase  20.6% increase |
| Gong *et al.* 2014 | Daily average PM_2.5_ (not reported)  Daily average UFP (10.6±4.3 10^3^/cm^3^) | FeNO  FeNO | 40.7% increase  25.3% increase |
| Chen *et al.* 2015 | 2 hr average PM (≤ 74.19±3.46 μg/m^3^)  2 hr average BC (≤ 11.54±0.57 μg/m^3^) | FeNO  FeNO | Increase  Increase |
| Barath *et al.* 2013 | Hourly average PM_10_ (300 μg/m^3^) | FeNO | Increase |
| Strak *et al.* 2012 | 5 hr average PM_2.5_ (39 (8-167) μg/m^3^)  5 hr average PNC (23.0 (7.0-74.7) 10^3^/cm^3^) | FeNO  FeNO | No effect  11.0% increase |
| Mirowsky *et al.* 2015 | 2 hr average PM_2.5_ (≤ 31 (11-45) μg/m^3^)  2 hr average BC (≤ 7.2 (4.1-10.9) μg/m^3^) | FeNO  FeNO | No effect  No effect |
| Cui *et al.* 2018 | 13 hr average PM_2.5_ (≤ 33.2±10.8 μg/m^3^)  13 hr average PNC (≤ 5.9±2.5 10^3^/cm^3^) | FeNO, aortic pressure index  FeNO, aortic pressure index | No effect  No effect |
| Lucking *et al.* 2011 | Hourly average PM (320±10 μg/m^3^) | FeNO, aortic augmentation pressure, aortic pressure index | No effect |
| Byrd *et al.* 2016 | 2 hr average PM_2.5-10_ (164.1±80.4 μg/m^3^) | aortic augmentation pressure, aortic pressure index | No effect |
| Lundback *et al.* 2009 | Hourly average PM (330±12 μg/m^3^) | aortic augmentation pressure, aortic pressure index | Increase |
| Unosson *et al.* 2013 | 3 hr average PM_1_ (314±38 μg/m^3^) | aortic augmentation pressure, aortic pressure index | Increases |
| Lenters *et al.* 2010 | Annual average PM_2.5_ (20.7±1.2 μg/m^3^) | aortic pressure index | No effect |
| Bard *et al.* 2010 | Hourly average PM_2.5_ (315±116 μg/m^3^) | aortic augmentation pressure, aortic pressure index, ejection duration | No effect |
| Mahmud *et al.* 2004 | Secondhand smoke vs. room air  Secondhand smoke vs. room air | aortic pressure index  ejection duration | Increases  No effect |

Abbreviation: FeNO, fractional exhaled nitric oxide; PNC, particle number concentration; UFP, ultrafine particles; BC: black carbon.


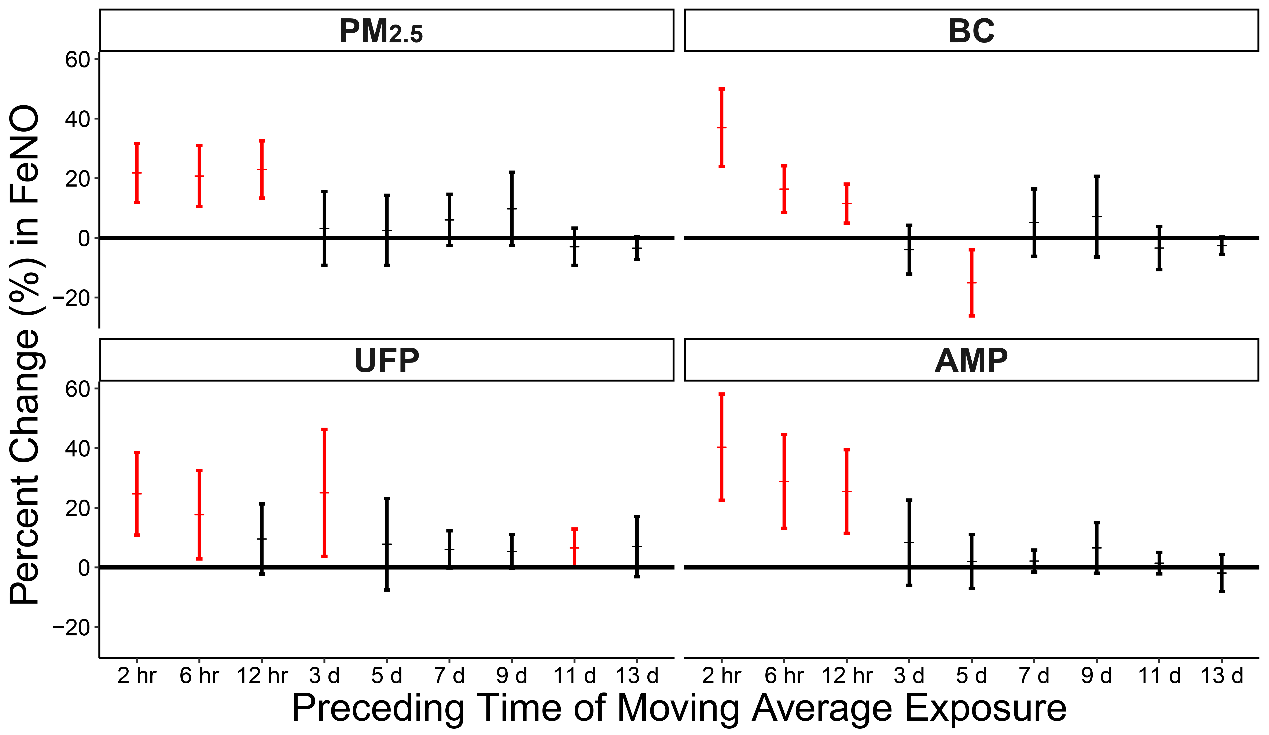


**Figure S1 percent changes in FeNO per IQR increases in PM_2.5_ and its constituents**. All models are adjusted for ambient temperature and relative humidity. The x-axis denotes the time window of exposure to PM_2.5_ and its constituents. The error bars denote the 95% CI of β, and the red ones denote the significant association between particle exposure and the level of FeNO. The bold horizontals denote the null association.


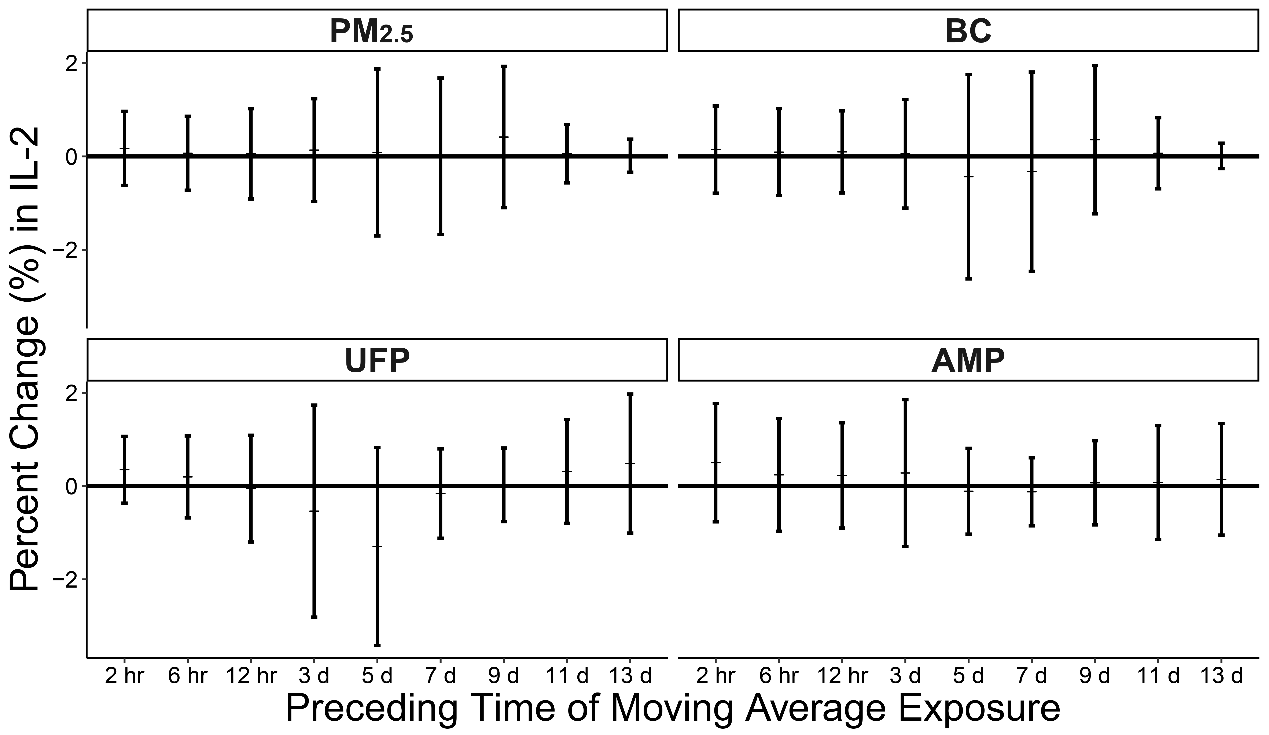


**Figure S2 percent changes in IL-2 per IQR increases in PM_2.5_ and its constituents.** All models are adjusted for ambient temperature and relative humidity. The x-axis denotes the time window of exposure to PM_2.5_ and its constituents. The error bars denote the 95% CI of β, and the red ones denote the significant association between particle exposure and the level of IL-2. The bold horizontals denote the null association.

**
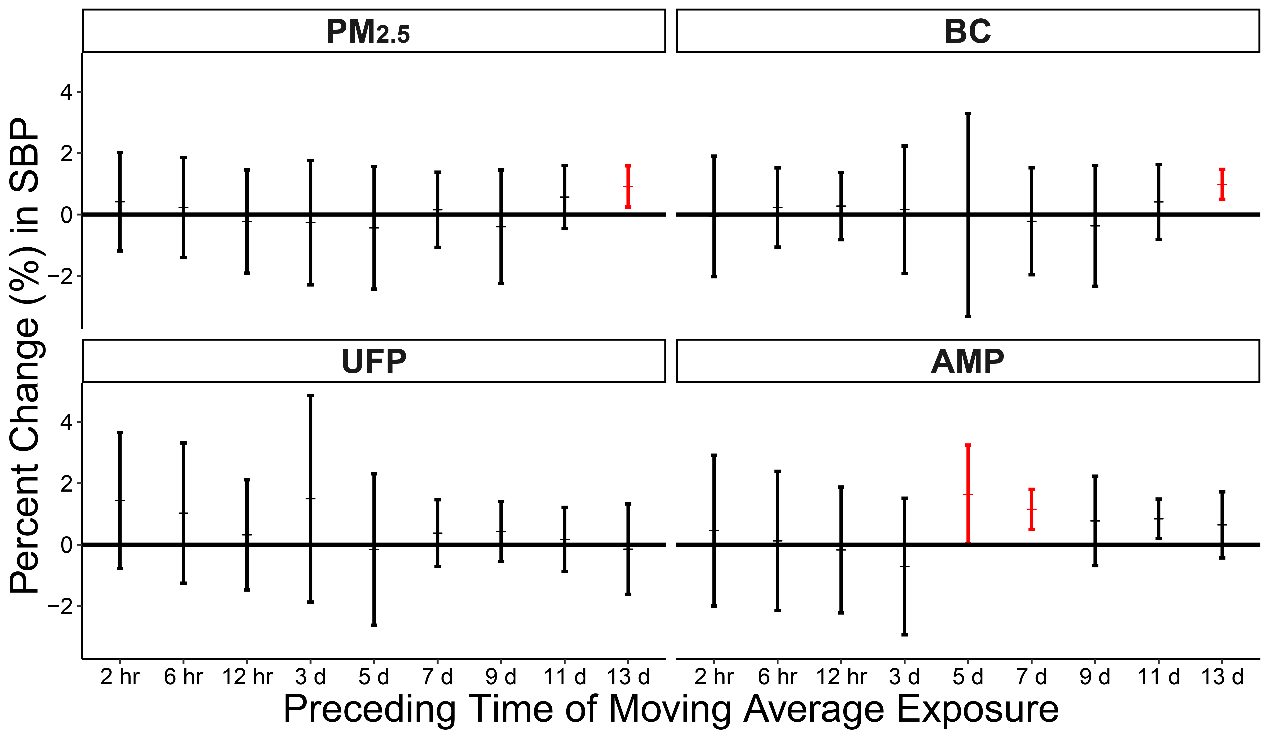
Figure S3 percent changes in SBP per IQR increases in PM_2.5_ and its constituents.** All models are adjusted for ambient temperature and relative humidity. The x-axis denotes the time window of exposure to PM_2.5_ and its constituents. The error bars denote the 95% CI of β, and the red ones denote the significant association between particle exposure and the level of SBP. The bold horizontals denote the null association.


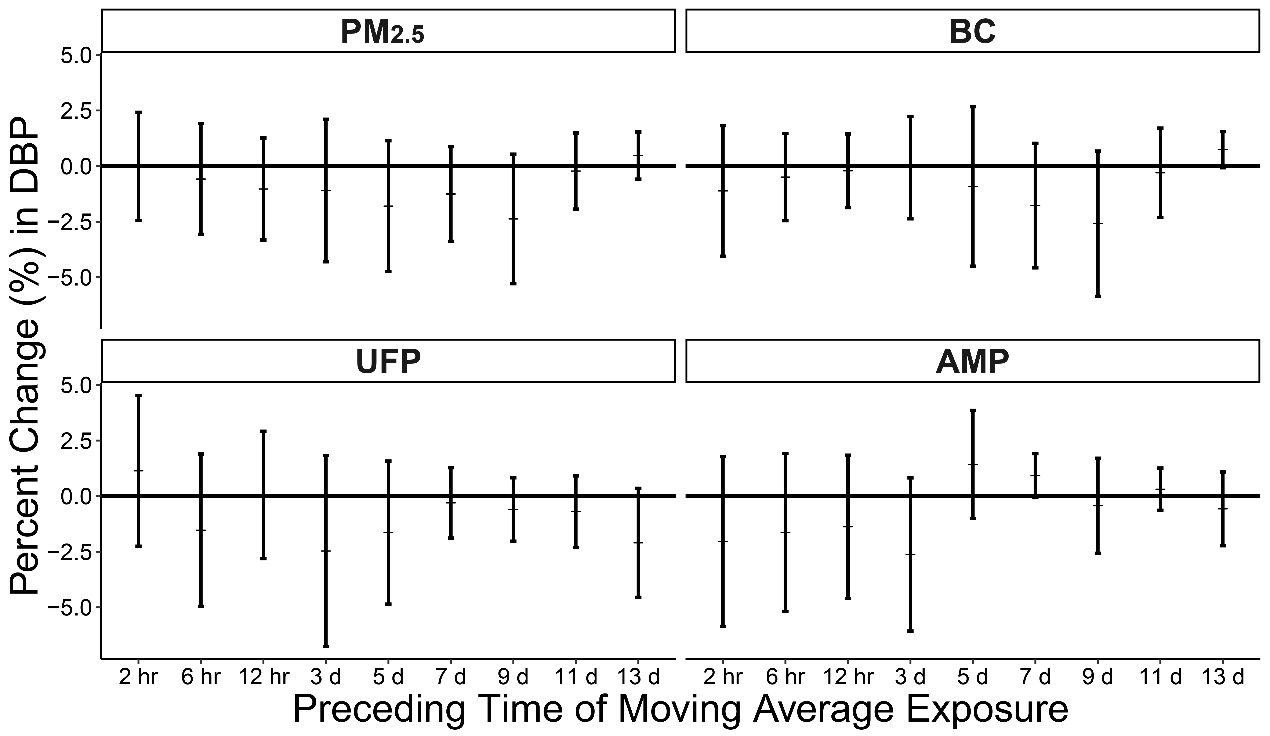


**Figure S4 percent changes in DBP per IQR increases in PM_2.5_ and its constituents.** All models are adjusted for ambient temperature and relative humidity. The x-axis denotes the time window of exposure to PM_2.5_ and its constituents. The error bars denote the 95% CI of β. The bold horizontals denote the null association.


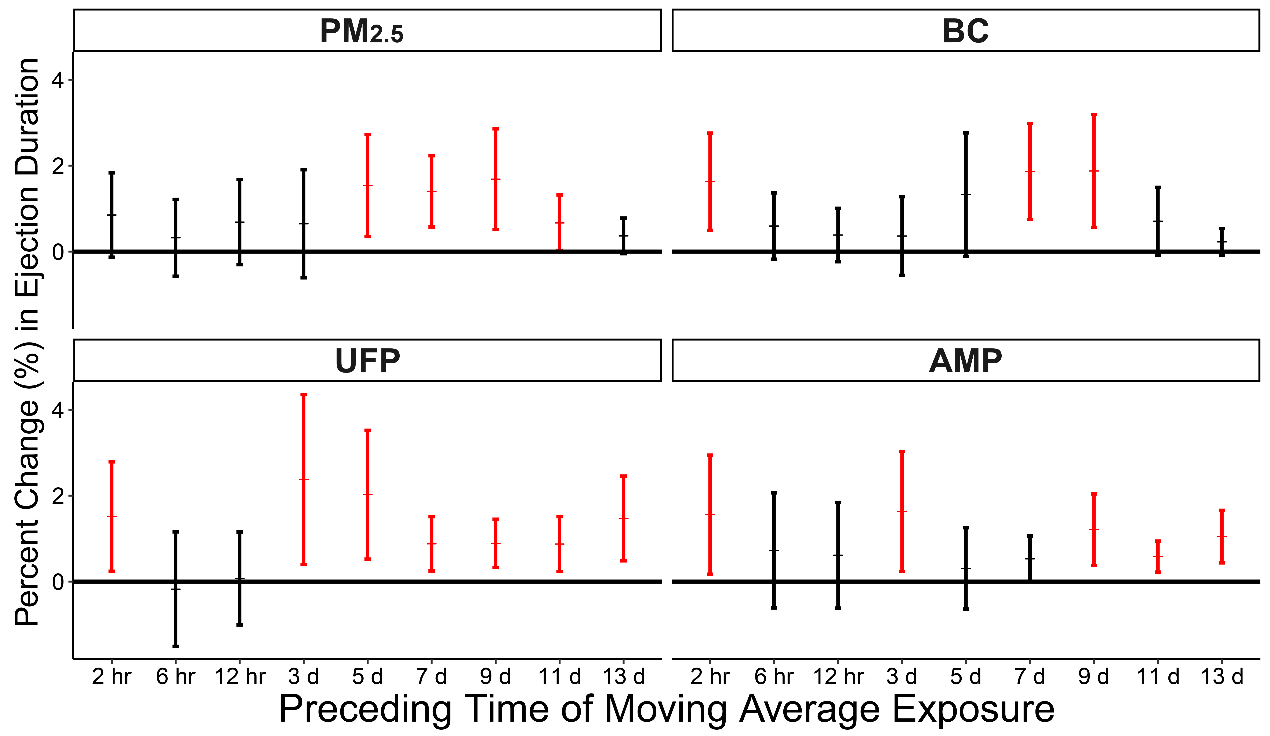


**Figure S5 percent changes in ejection duration per IQR increases in PM_2.5_ and its constituents**. All models are adjusted for ambient temperature and relative humidity. The x-axis denotes the time window of exposure to PM_2.5_ and its constituents. The error bars denote the 95% CI of β, and the red ones denote the significant association between particle exposure and the level of ejection duration. The bold horizontals denote the null association.


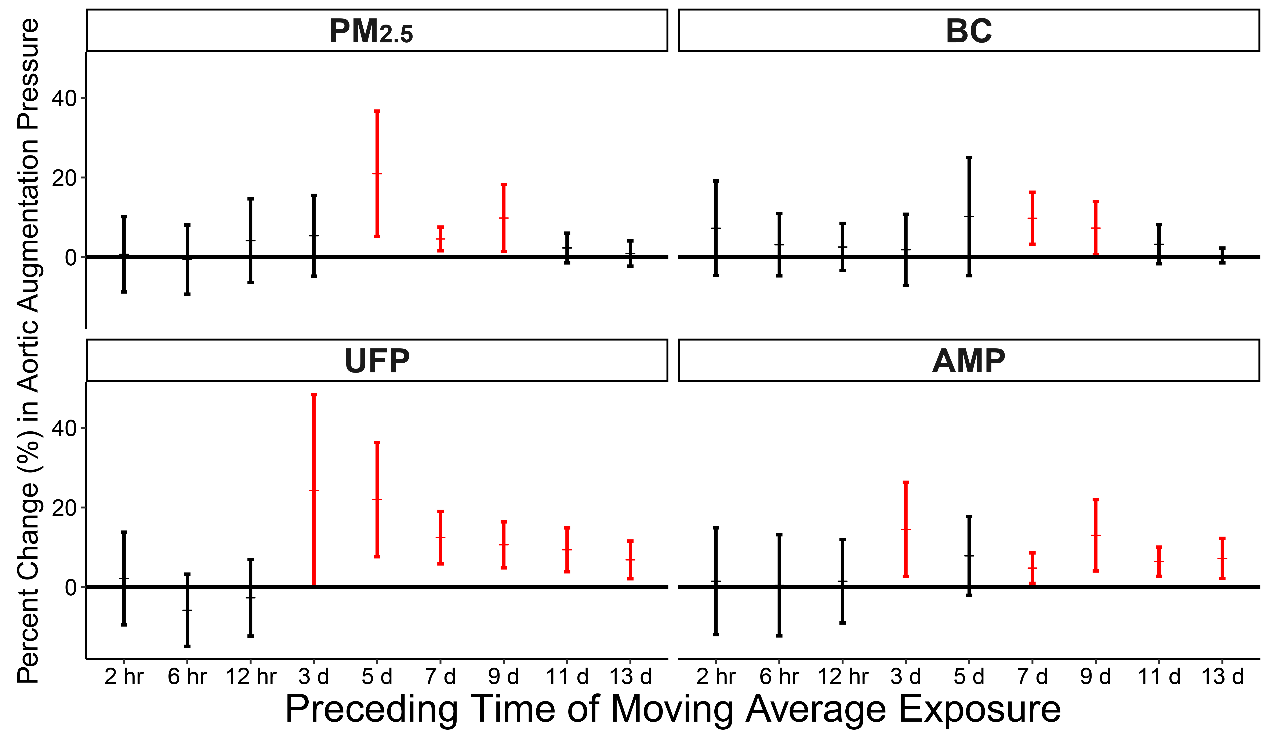


**Figure S6 percent changes in aortic augmentation pressure per IQR increases in PM_2.5_ and its constituents**. All models are adjusted for ambient temperature and relative humidity. The x-axis denotes the time window of exposure to PM_2.5_ and its constituents. The error bars denote the 95% CI of β, and the red ones denote the significant association between particle exposure and the level of aortic augmentation pressure. The bold horizontals denote the null association.


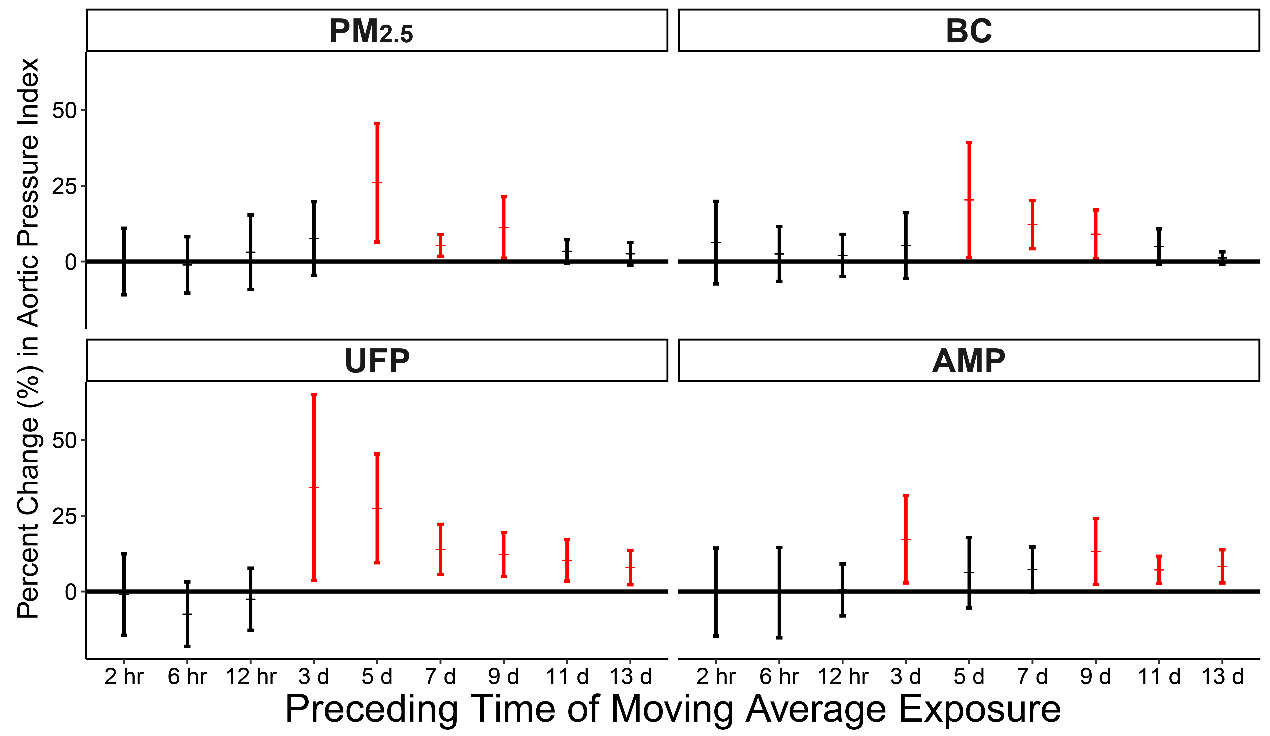


**Figure S7 percent changes in aortic pressure index per IQR increases in PM_2.5_ and its constituents**. All models are adjusted for ambient temperature and relative humidity. The x-axis denotes the time window of exposure to PM_2.5_ and its constituents. The error bars denote the 95% CI of β, and the red ones denote the significant association between particle exposure and the level of aortic pressure index. The bold horizontals denote the null association.


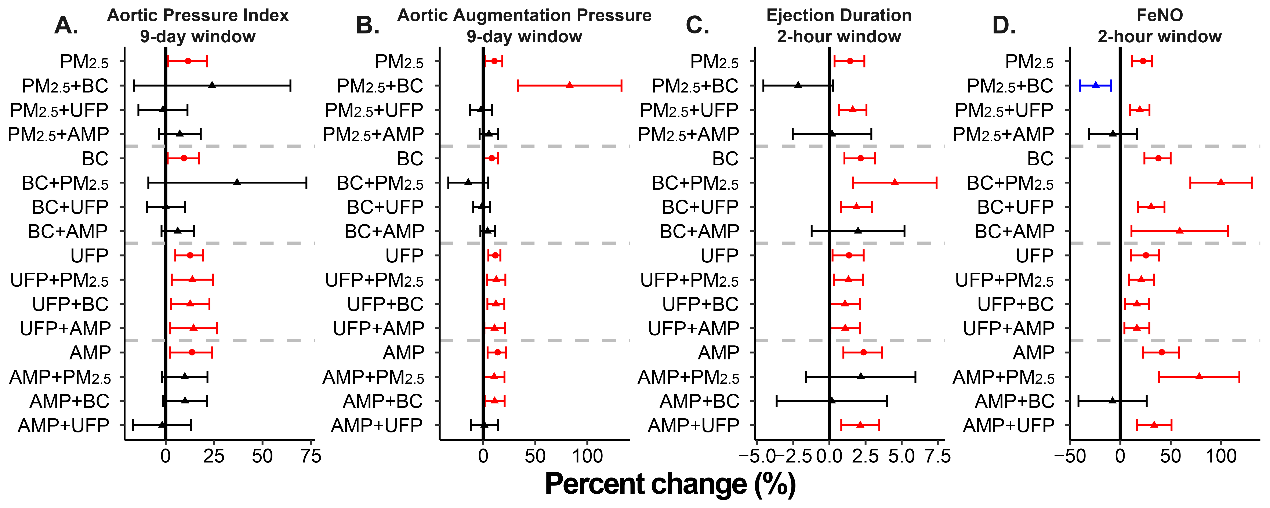


**Figure S8 percent changes in biomarkers per IQR increases in PM_2.5_ and its constituents in two-pollutant models.** A) aortic pressure index, B) aortic augmentation pressure, C) ejection duration, D) FeNO. All models are adjusted for ambient temperature and relative humidity. The circular and triangle dots denote the estimated changes from single-pollutant and two-pollutant models, respectively. The red and blue error bars denote the positive and negative association between particle exposure and the levels of the biomarkers, respectively.


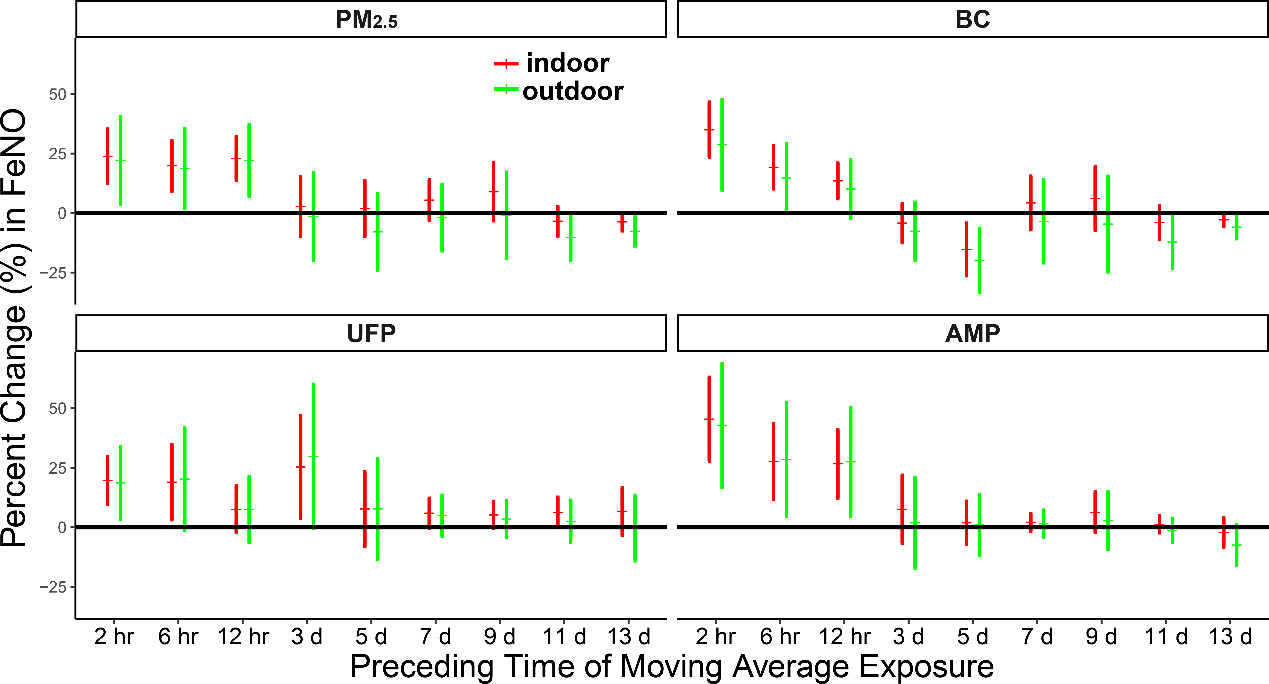


**Fig**ure **S9** **percent changes in FeNO associated with the IQR increases in concentrations of PM_2.5_ and its constituents stratified by exercise place**. All models are adjusted for ambient temperature and relative humidity. The red and green error bars denote the estimated changes in subjects who prefer indoor and outdoor exercise, respectively. ^★^ Denotes significant (*p*-value < 0.05) differences between the estimates.


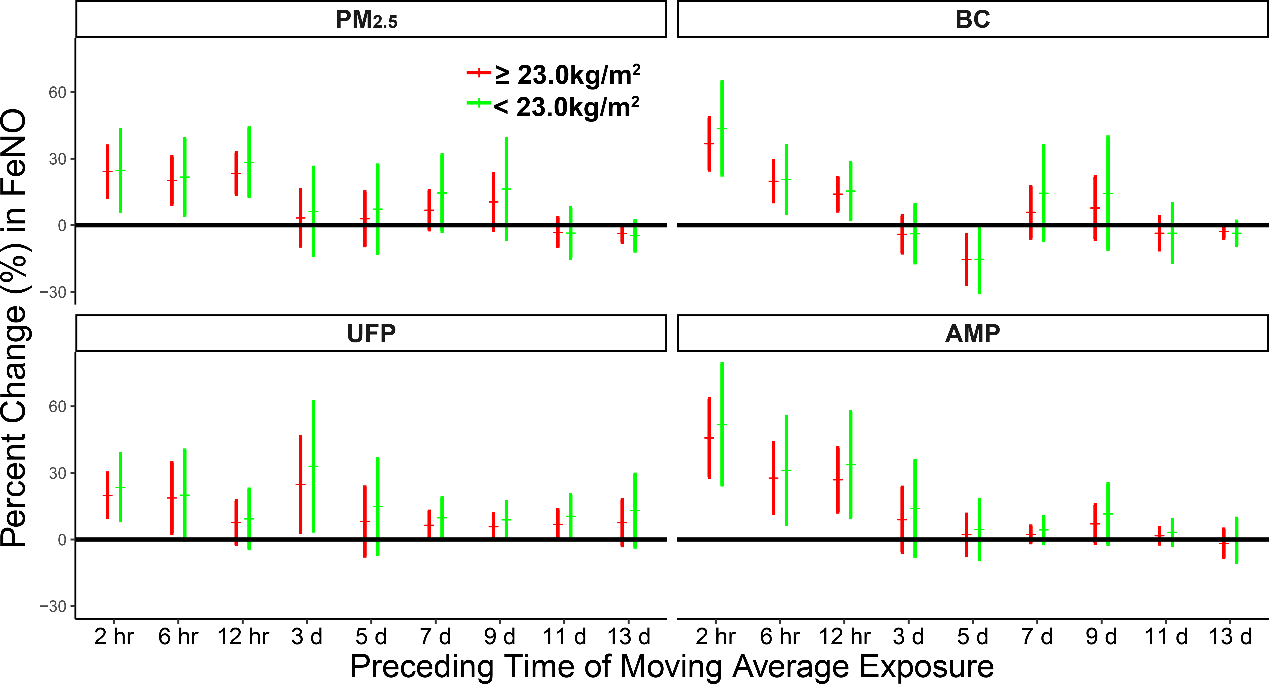


**Figure** **S10** **percent changes in FeNO associated with the IQR increases in concentrations of PM_2.5_ and its constituents stratified by BMI**. All models are adjusted for ambient temperature and relative humidity. The red and green error bars denote the estimated changes in subjects with BMI ≥ and < 23.0 kg/m^2^, respectively. ^★^ Denotes significant (*p*-value < 0.05) differences between the estimates.


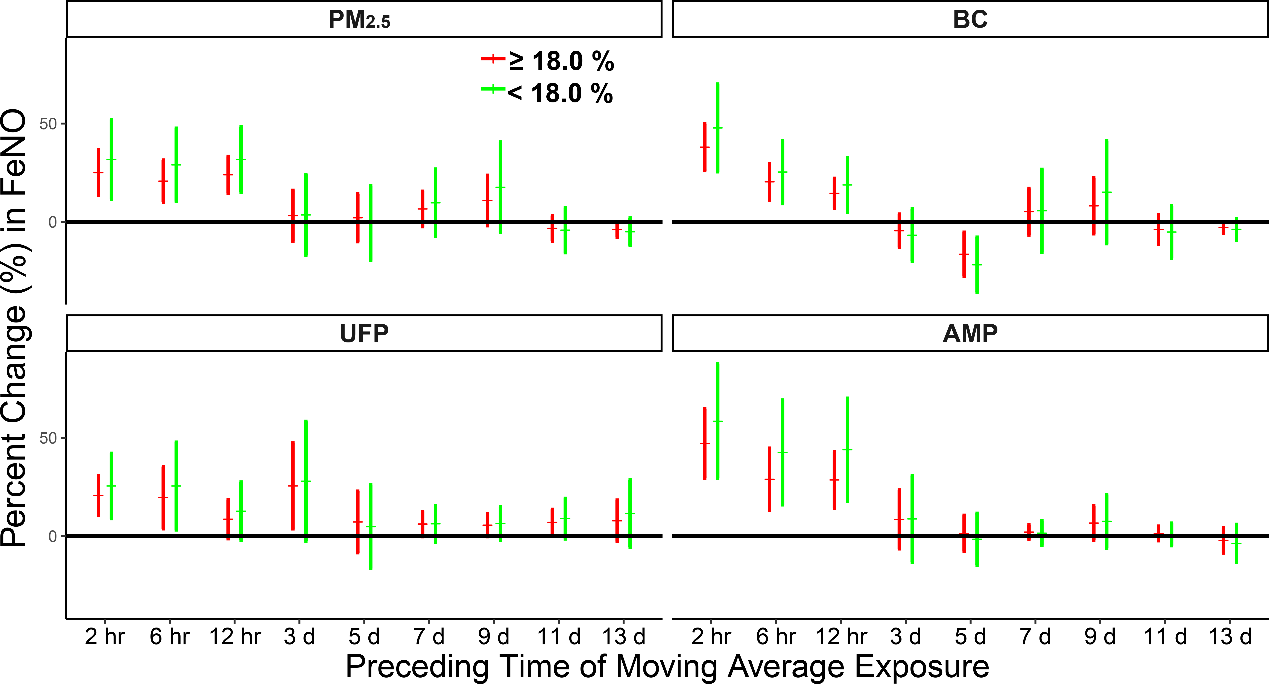


**Figure** **S11** **percent changes in FeNO associated with the IQR increases in concentrations of PM_2.5_ and its constituents stratified by BFR**. All models are adjusted for ambient temperature and relative humidity. The red and green error bars denote the estimated changes in subjects with BFR ≥ and < 18.0 %, respectively. ^★^ Denotes significant (*p*-value < 0.05) differences between the estimates.


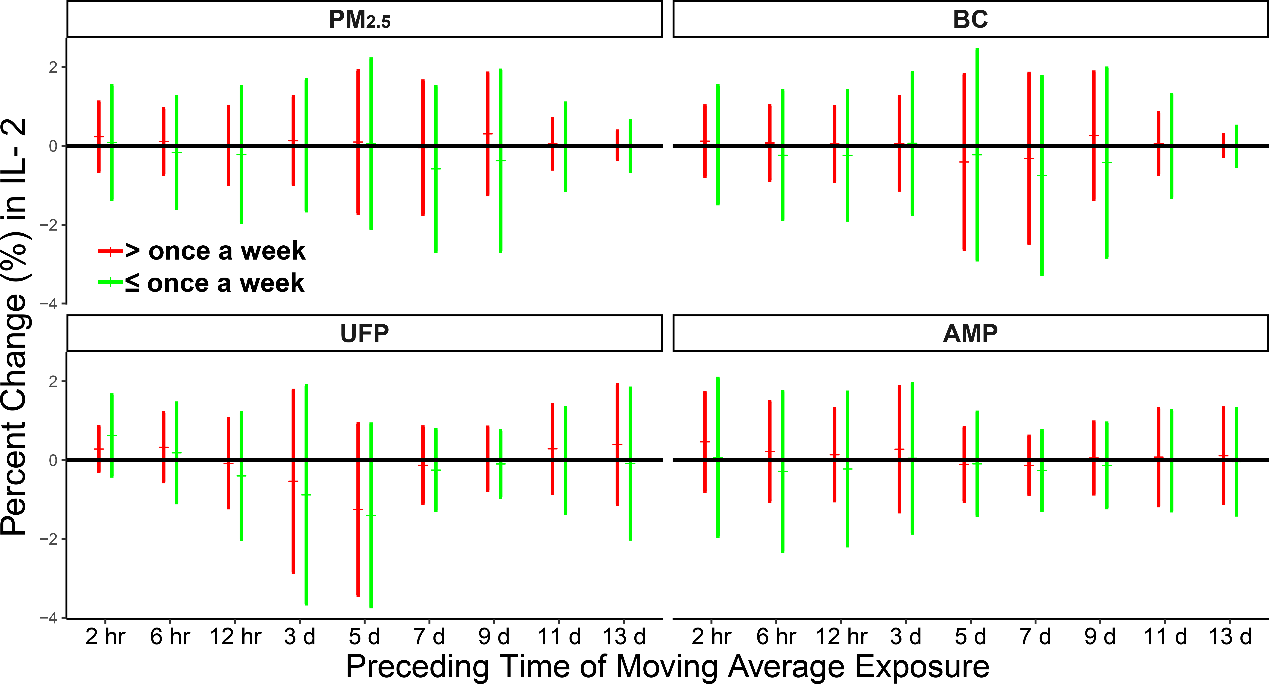


**Figure** **S12** **percent changes in IL-2 associated with the IQR increases in concentrations of PM_2.5_ and its constituents stratified by exercise frequency**. All models are adjusted for ambient temperature and relative humidity. The red and green error bars denote the estimated changes in subjects who exercise more and no more than once a week, respectively. ^★^ Denotes significant (*p*-value < 0.05) differences between the estimates.


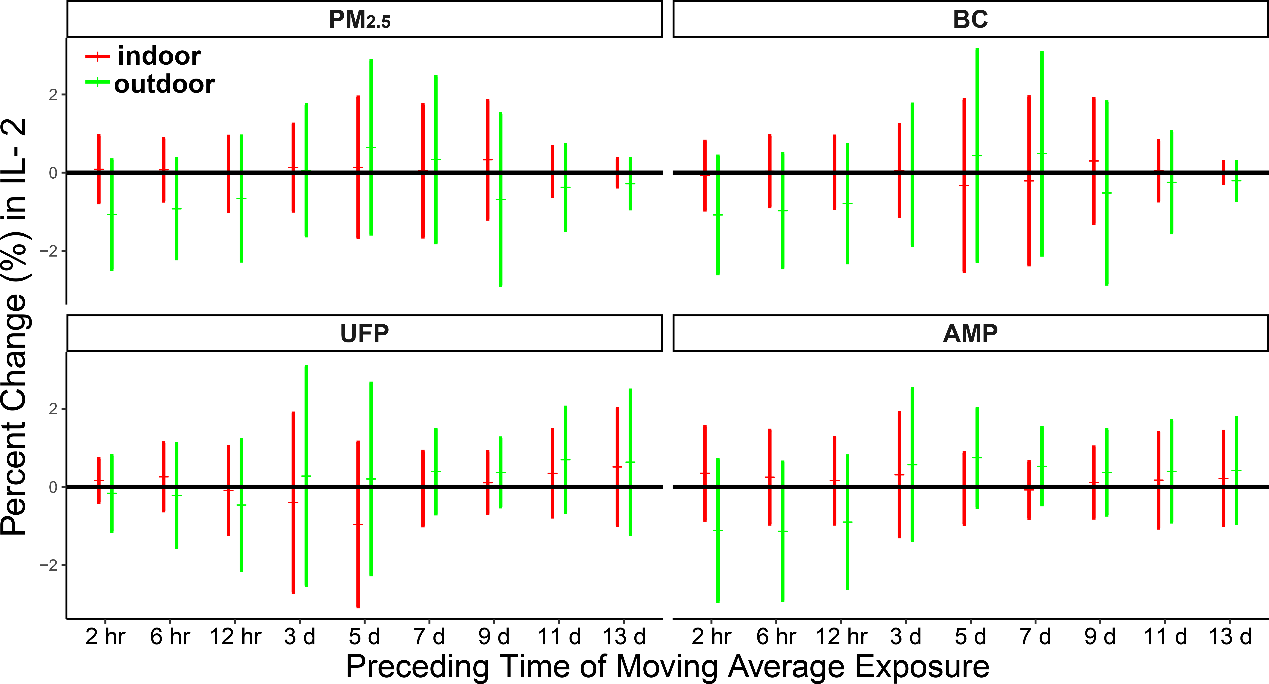


**Figure** **S13** **percent changes in IL-2 associated with the IQR increases in concentrations of PM_2.5_ and its constituents stratified by exercise place**. All models are adjusted for ambient temperature and relative humidity. The red and green error bars denote the estimated changes in subjects who prefer indoor and outdoor exercise, respectively. ^★^ Denotes significant (*p*-value < 0.05) differences between the estimates.


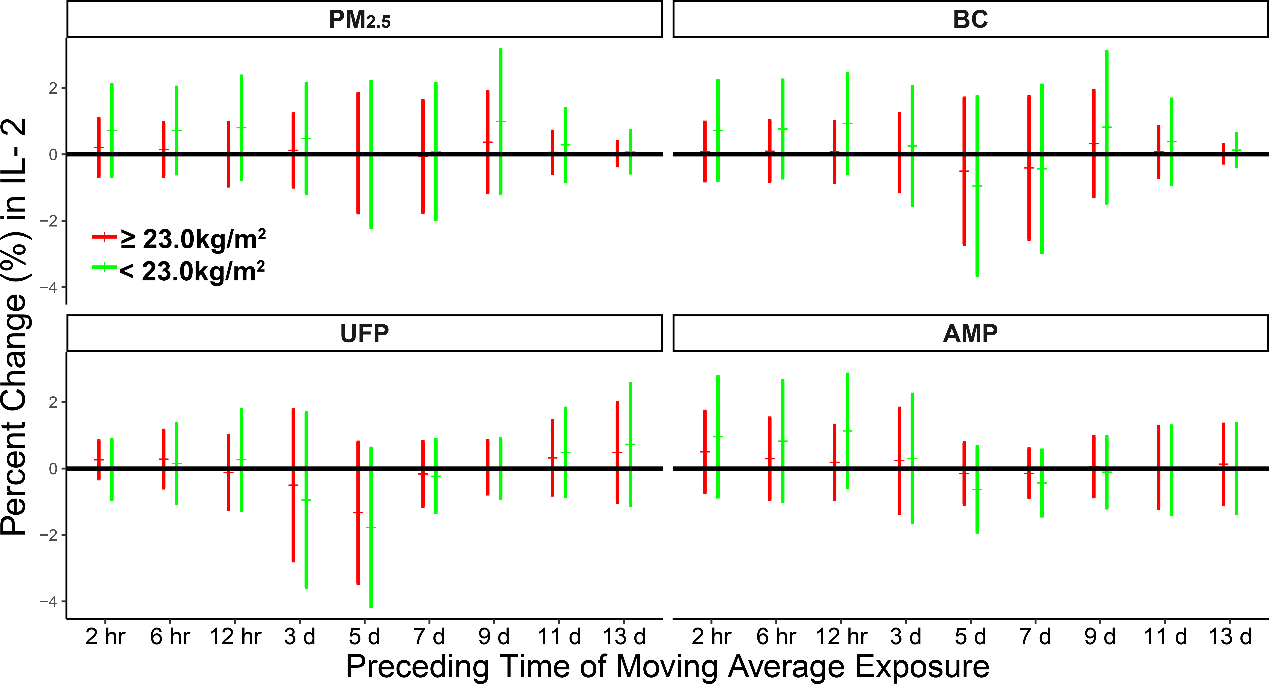


**Figure** **S14** **percent changes in IL-2 associated with the IQR increases in concentrations of PM_2.5_ and its constituents stratified by BMI**. All models are adjusted for ambient temperature and relative humidity. The red and green error bars denote the estimated changes in subjects with BMI ≥ and < 23.0 kg/m^2^, respectively. ^★^ Denotes significant (*p*-value < 0.05) differences between the estimates.


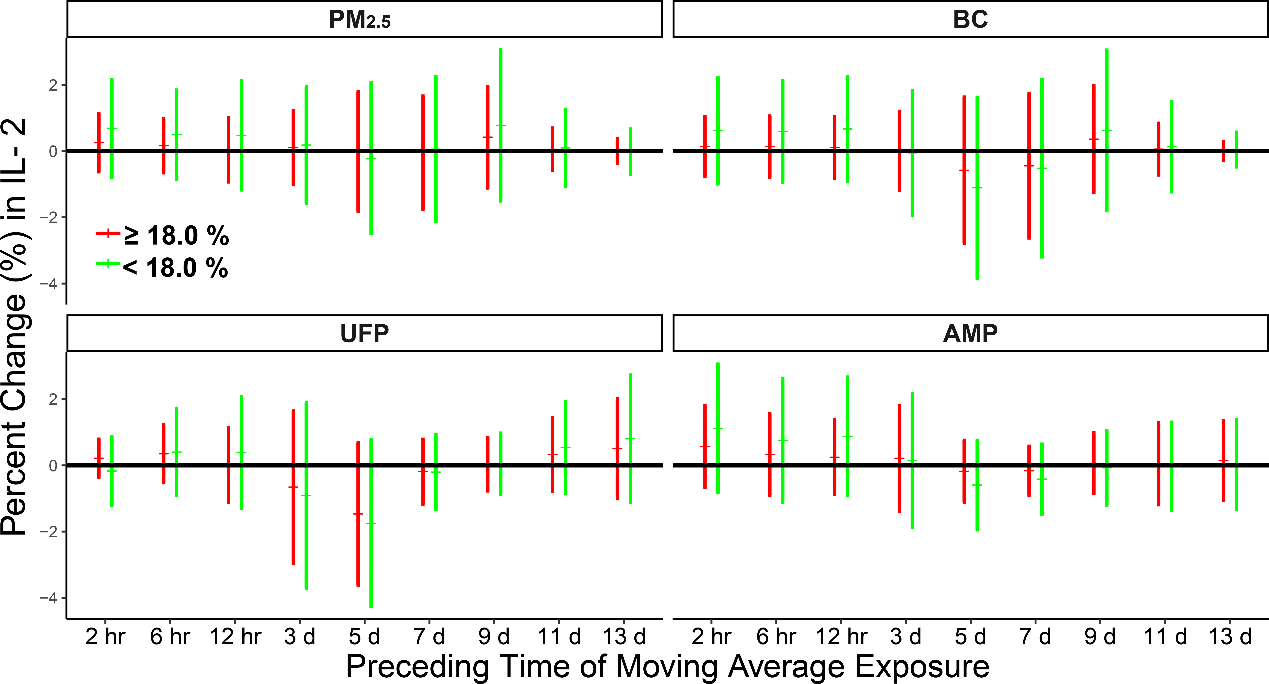


**Figure** **S15** **percent changes in IL-2 associated with the IQR increases in concentrations of PM_2.5_ and its constituents stratified by BFR**. All models are adjusted for ambient temperature and relative humidity. The red and green error bars denote the estimated changes in subjects with BFR ≥ and < 18.0 %, respectively. ^★^ Denotes significant (*p*-value < 0.05) differences between the estimates.


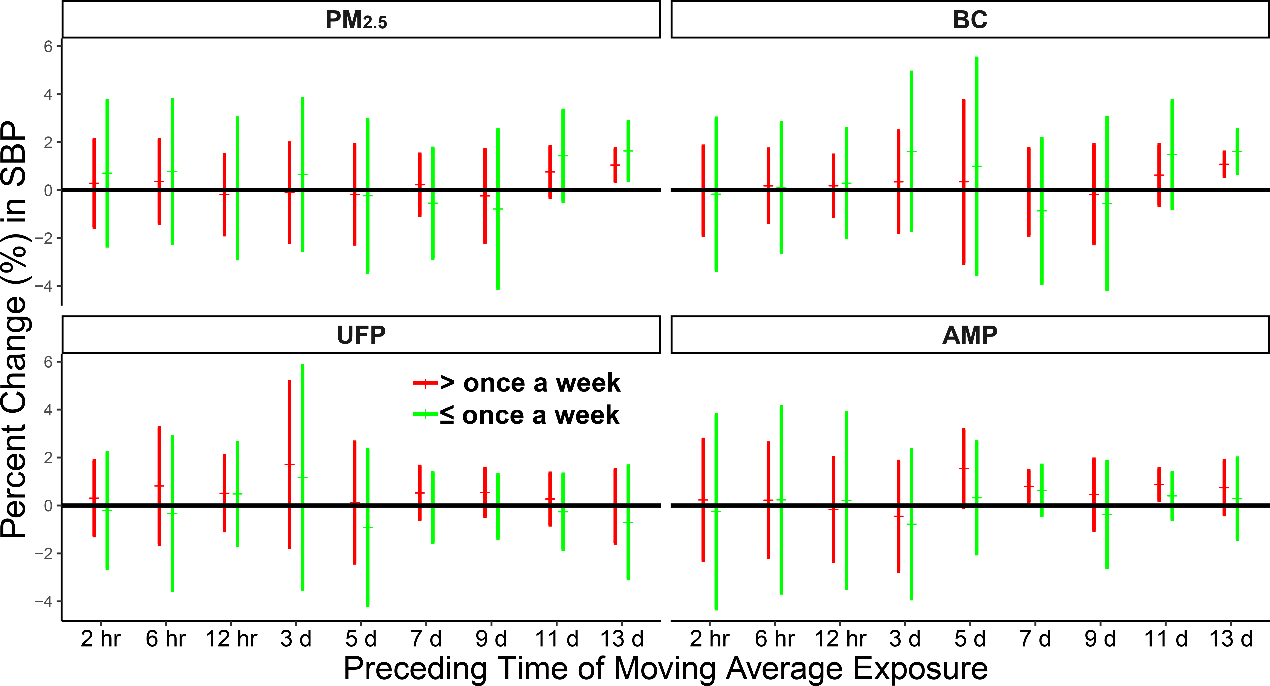


**Figure** **S16** **percent changes in SBP associated with the IQR increases in concentrations of PM_2.5_ and its constituents stratified by exercise frequency**. All models are adjusted for ambient temperature and relative humidity. The red and green error bars denote the estimated changes in subjects who exercise more and no more than once a week, respectively. ^★^ Denotes significant (*p*-value < 0.05) differences between the estimates.


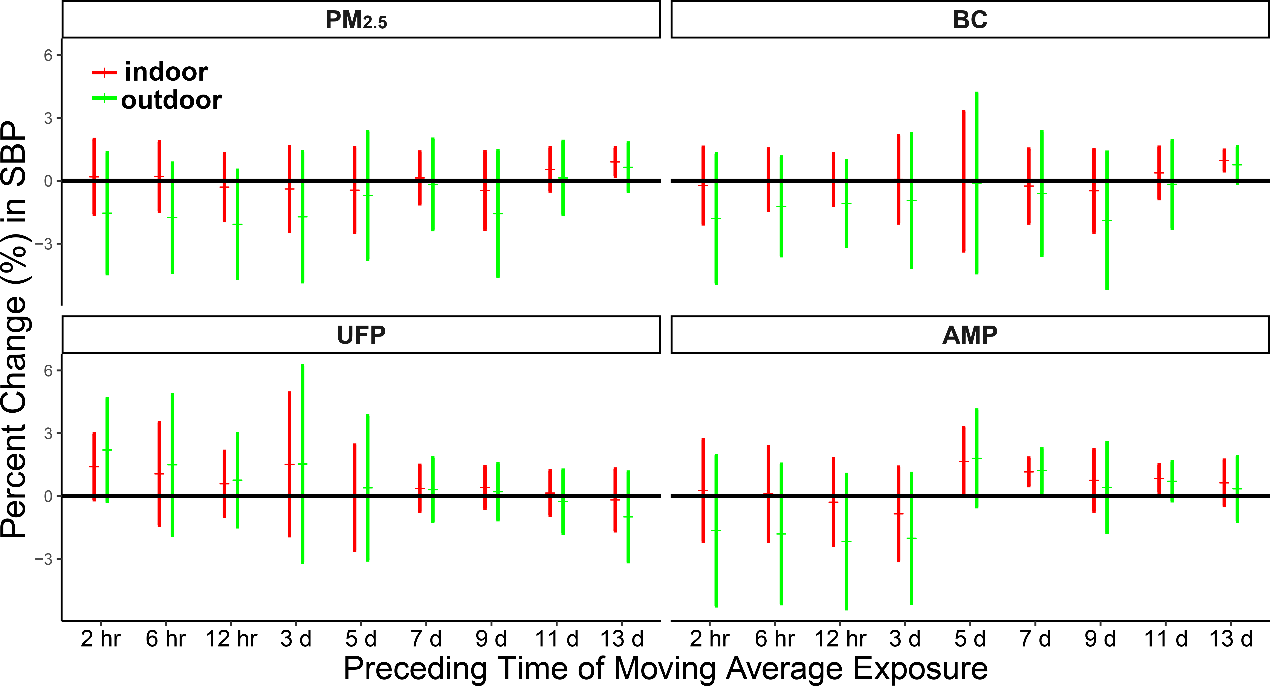


**Figure** **S17** **percent changes in SBP associated with the IQR increases in concentrations of PM_2.5_ and its constituents stratified by exercise place**. All models are adjusted for ambient temperature and relative humidity. The red and green error bars denote the estimated changes in subjects who prefer exercise indoor and out door, respectively. ^★^ Denotes significant (*p*-value < 0.05) differences between the estimates.


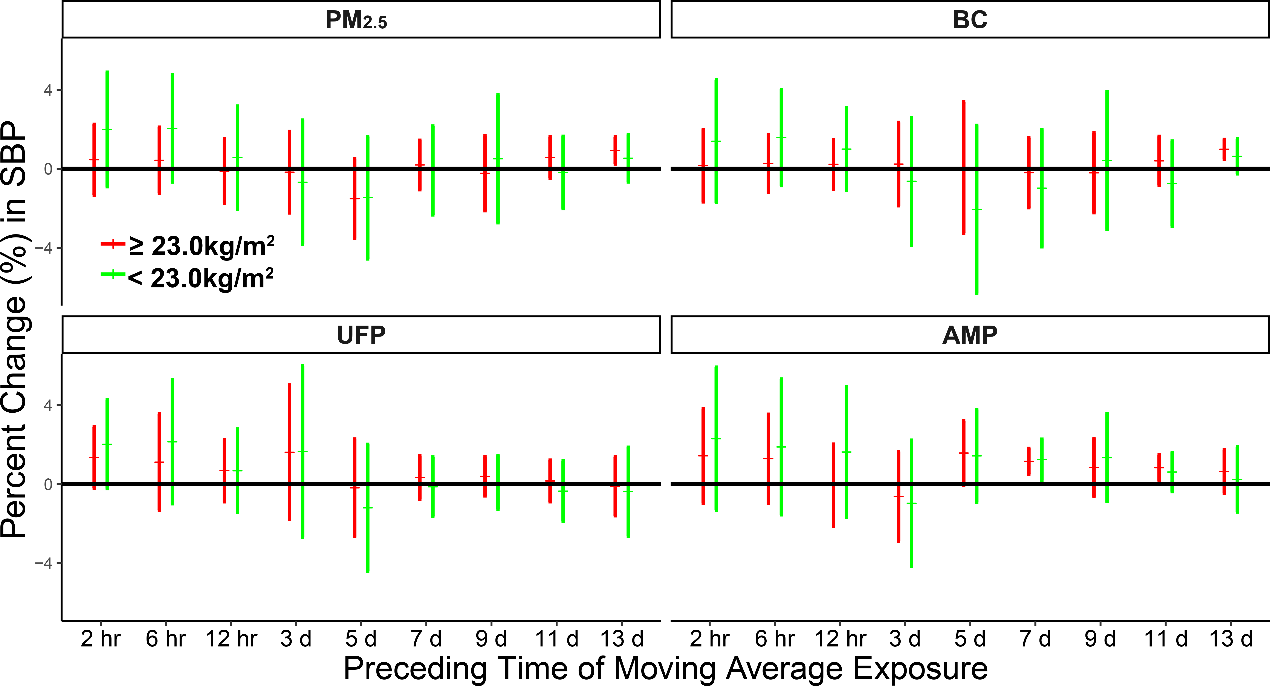


**Figure** **S18** **percent changes in SBP associated with the IQR increases in concentrations of PM_2.5_ and its constituents stratified by BMI**. All models are adjusted for ambient temperature and relative humidity. The red and green error bars denote the estimated changes in subjects with BMI ≥ and < 23.0 kg/m^2^, respectively. ^★^ Denotes significant (*p*-value < 0.05) differences between the estimates.


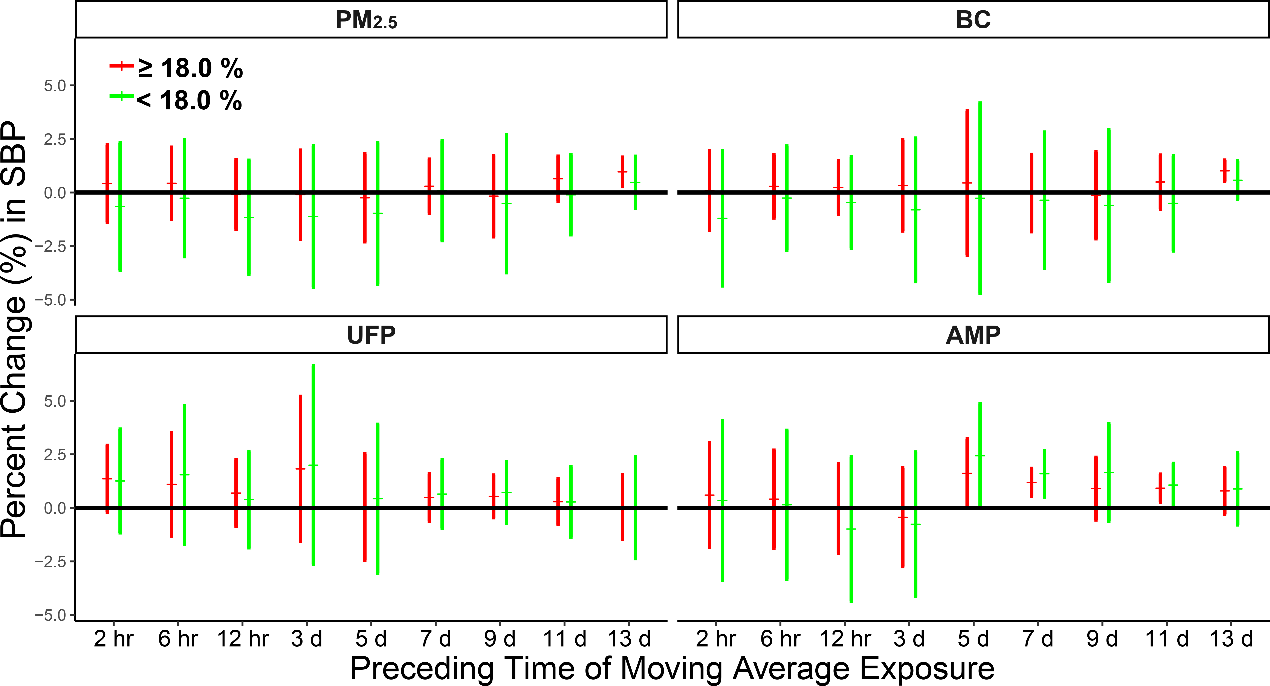


**Figure S19** **percent changes in SBP associated with the IQR increases in concentrations of PM_2.5_ and its constituents stratified by BFR**. All models are adjusted for ambient temperature and relative humidity. The red and green error bars denote the estimated changes in subjects with BFR ≥ and < 18.0 %, respectively. ^★^ Denotes significant (*p*-value < 0.05) differences between the estimates.


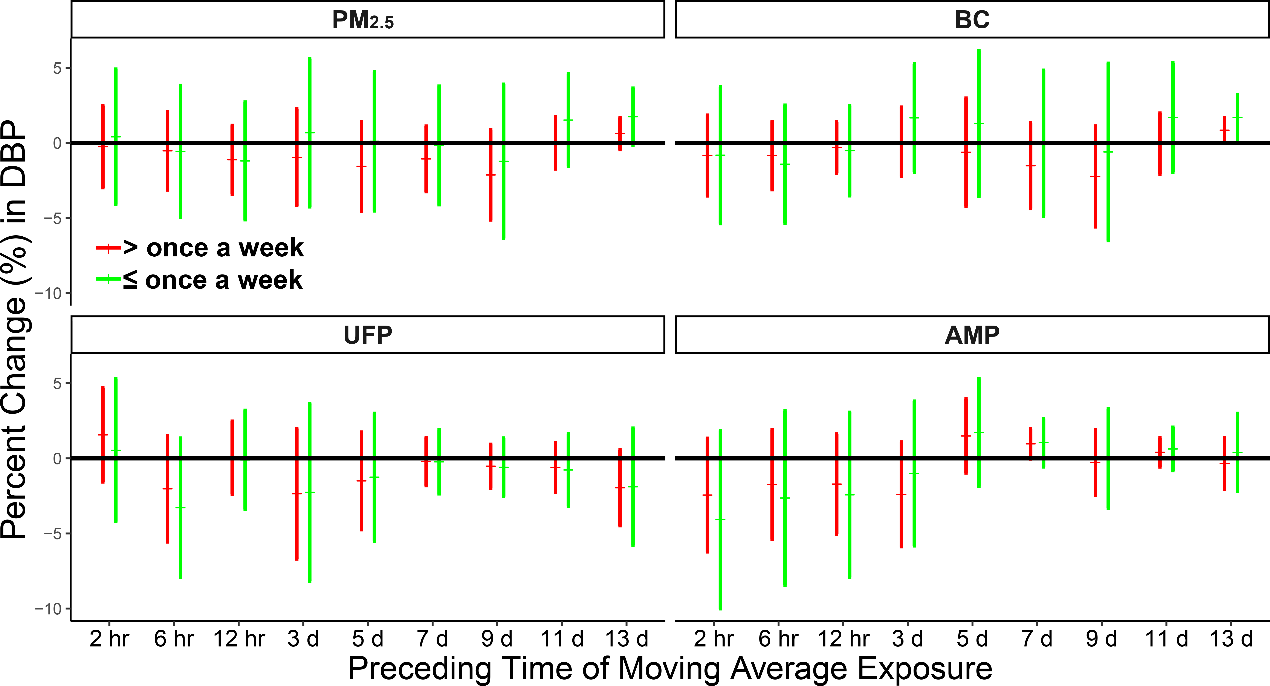


**Figure** **S20** **percent changes in DBP associated with the IQR increases in concentrations of PM_2.5_ and its constituents stratified by exercise frequency**. All models are adjusted for ambient temperature and relative humidity. The red and green error bars denote the estimated changes in subjects who exercise more and no more than once a week, respectively. ^★^ Denotes significant (*p*-value < 0.05) differences between the estimates.


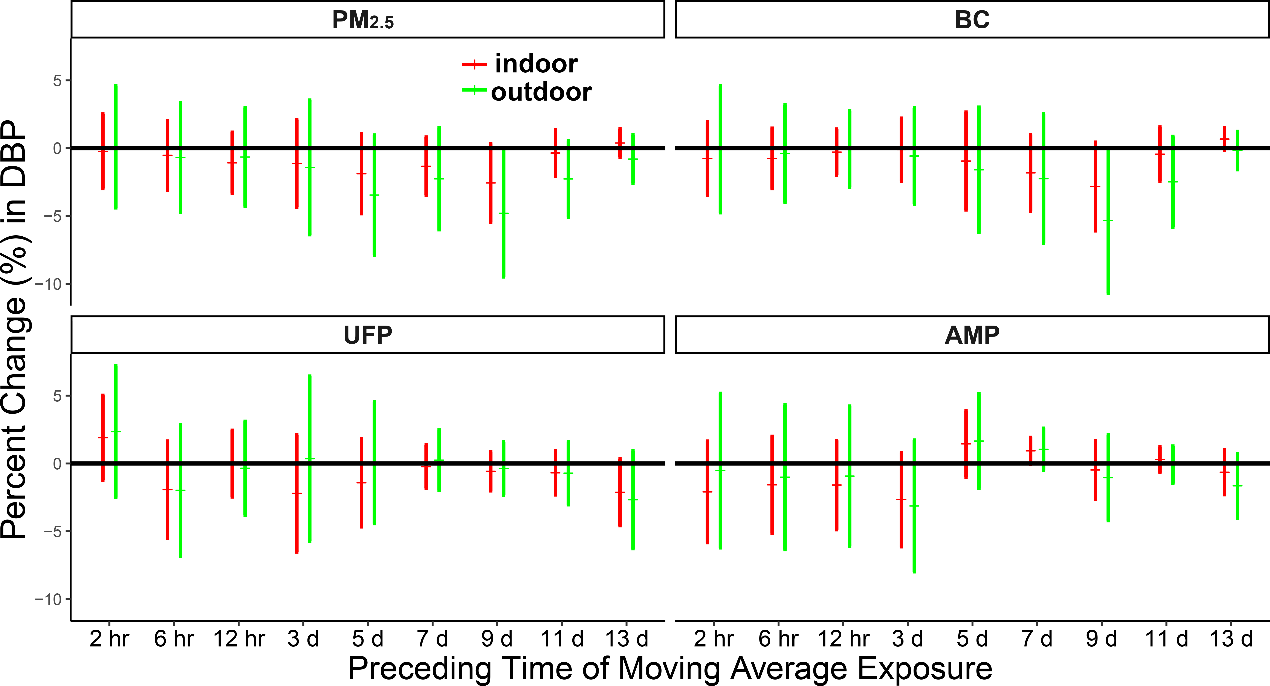


**Figure** **S21** **percent changes in DBP associated with the IQR increases in concentrations of PM_2.5_ and its constituents stratified by exercise place**. All models are adjusted for ambient temperature and relative humidity. The red and green error bars denote the estimated changes in subjects who prefer exercise indoor and out door, respectively. ^★^ Denotes significant (*p*-value < 0.05) differences between the estimates.


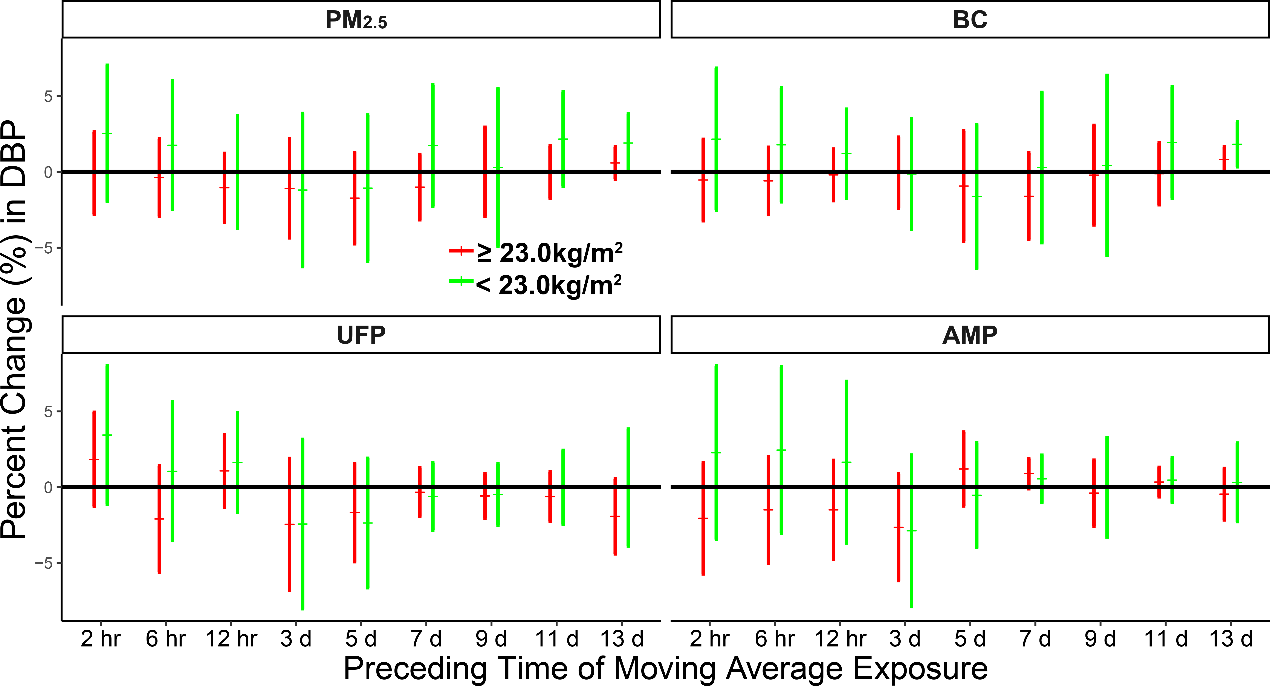


**Figure** **S22** **percent changes in DBP associated with the IQR increases in concentrations of PM_2.5_ and its constituents stratified by BMI**. All models are adjusted for ambient temperature and relative humidity. The red and green error bars denote the estimated changes in subjects with BMI ≥ and < 23.0 kg/m^2^, respectively. ^★^ Denotes significant (*p*-value < 0.05) differences between the estimates.


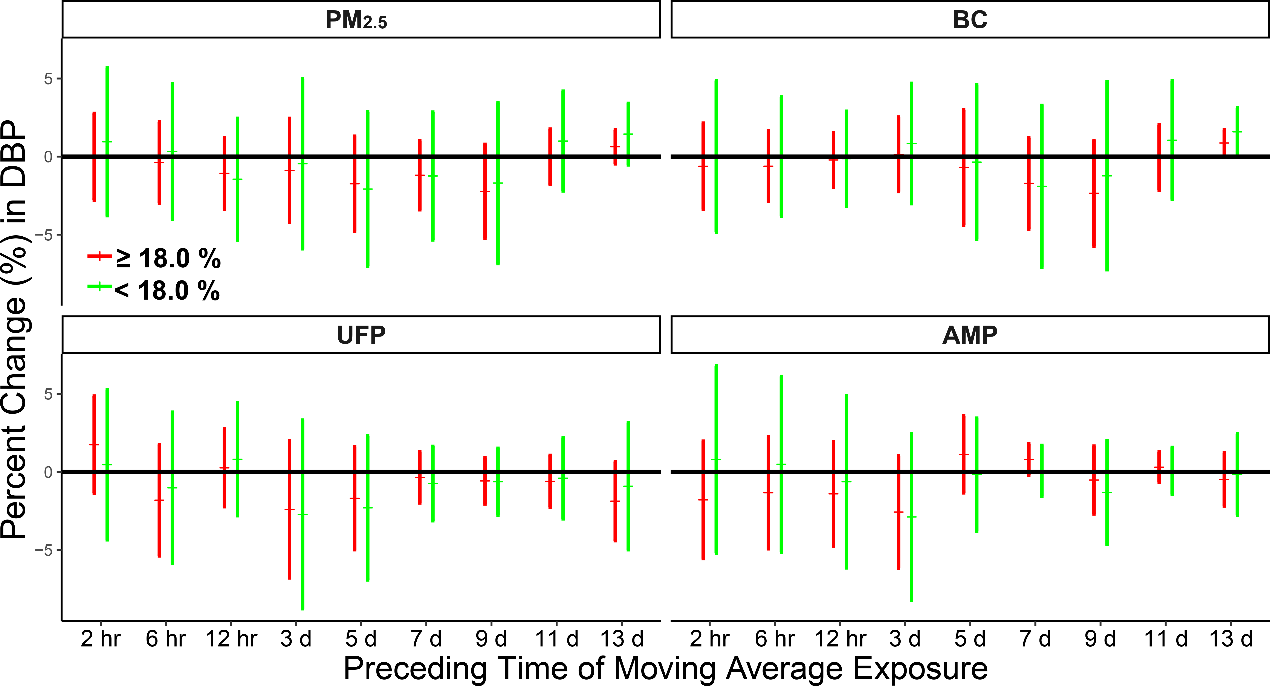


**Figure** **S23** **percent changes in DBP associated with the IQR increases in concentrations of PM_2.5_ and its constituents stratified by BFR**. All models are adjusted for ambient temperature and relative humidity. The red and green error bars denote the estimated changes in subjects with BFR ≥ and < 18.0 %, respectively. ^★^ Denotes significant (*p*-value < 0.05) differences between the estimates.


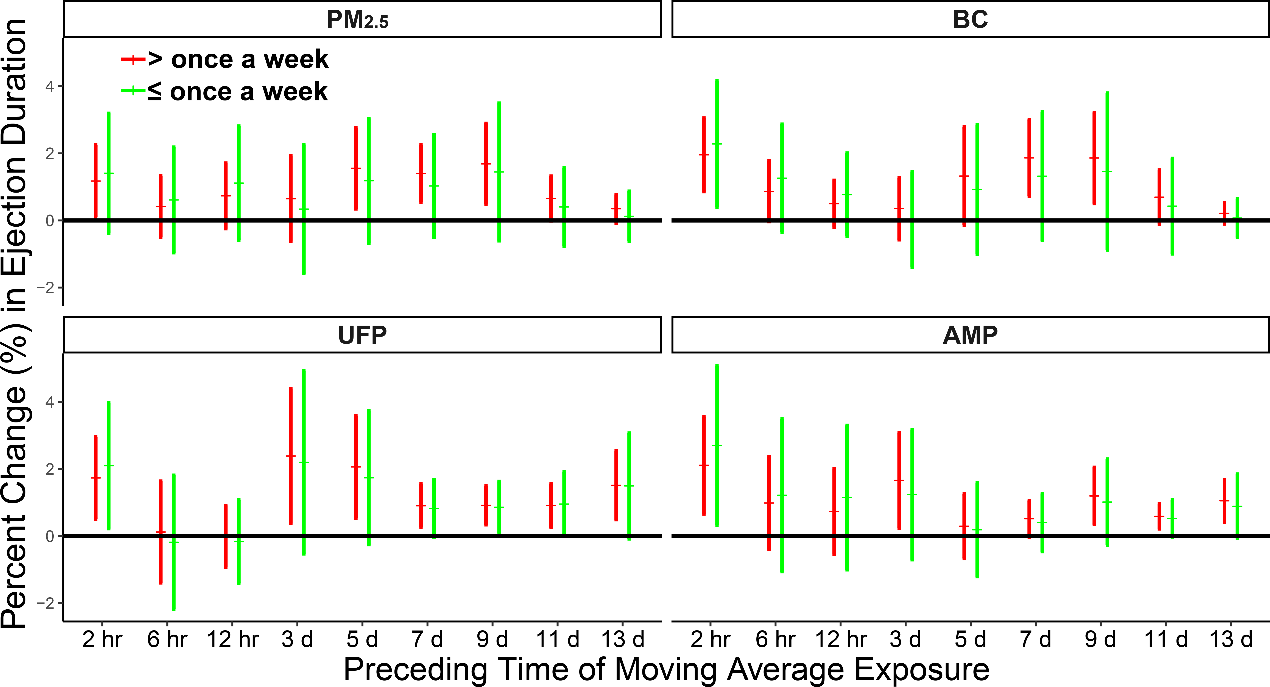


**Figure** **S24** **percent changes in ejection duration associated with the IQR increases in concentrations of PM_2.5_ and its constituents stratified by exercise frequency**. All models are adjusted for ambient temperature and relative humidity. The red and green error bars denote the estimated changes in subjects who exercise more and no more than once a week, respectively. ^★^ Denotes significant (*p*-value < 0.05) differences between the estimates.


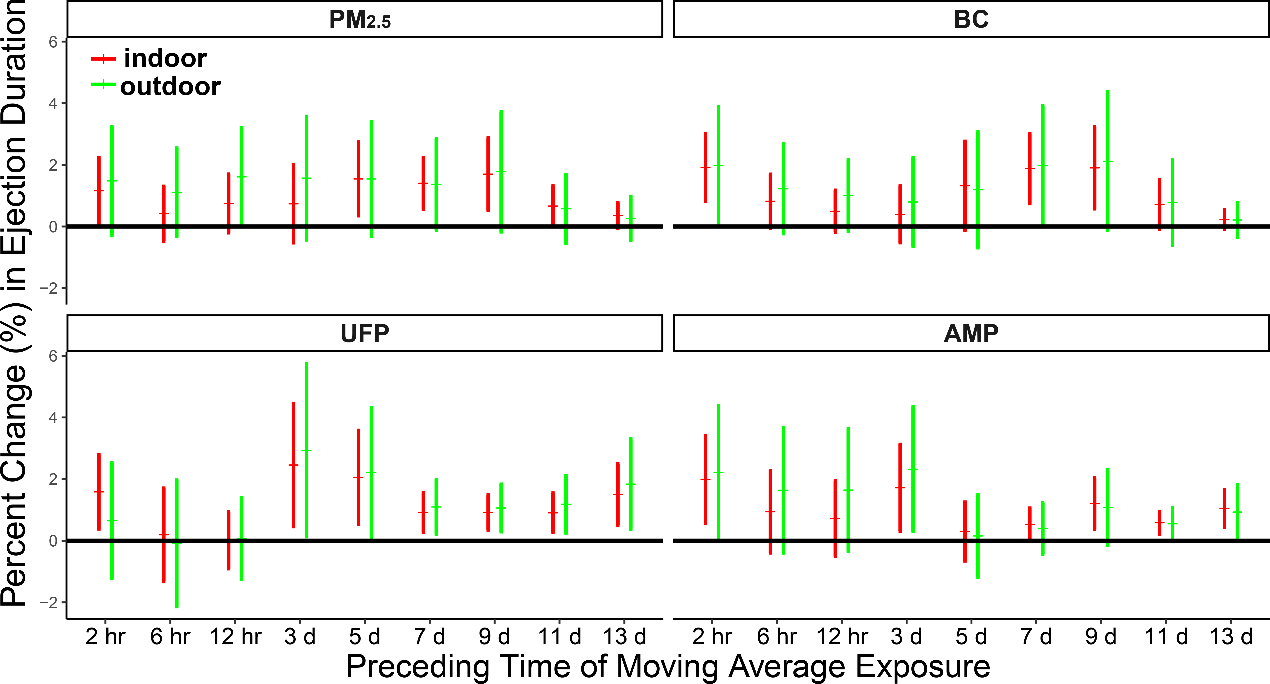


**Figure** **S25** **percent changes in ejection duration associated with the IQR increases in concentrations of PM_2.5_ and its constituents stratified by exercise place**. All models are adjusted for ambient temperature and relative humidity. The red and green error bars denote the estimated changes in subjects who prefer indoor and outdoor exercise, respectively. ^★^ Denotes significant (*p*-value < 0.05) differences between the estimates.


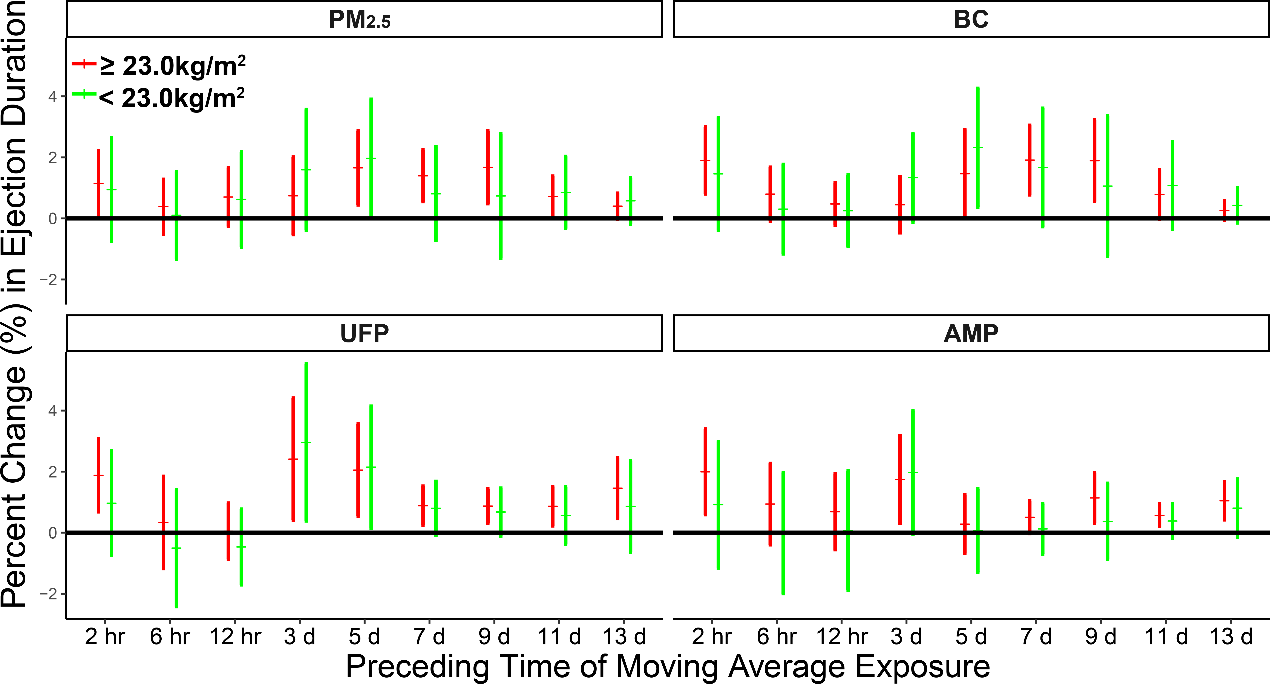


**Figure** **S26** **percent changes in ejection duration associated with the IQR increases in concentrations of PM_2.5_ and its constituents stratified by BMI**. All models are adjusted for ambient temperature and relative humidity. The red and green error bars denote the estimated changes in subjects with BMI ≥ and < 23.0 kg/m^2^, respectively. ^★^ Denotes significant (*p*-value < 0.05) differences between the estimates.


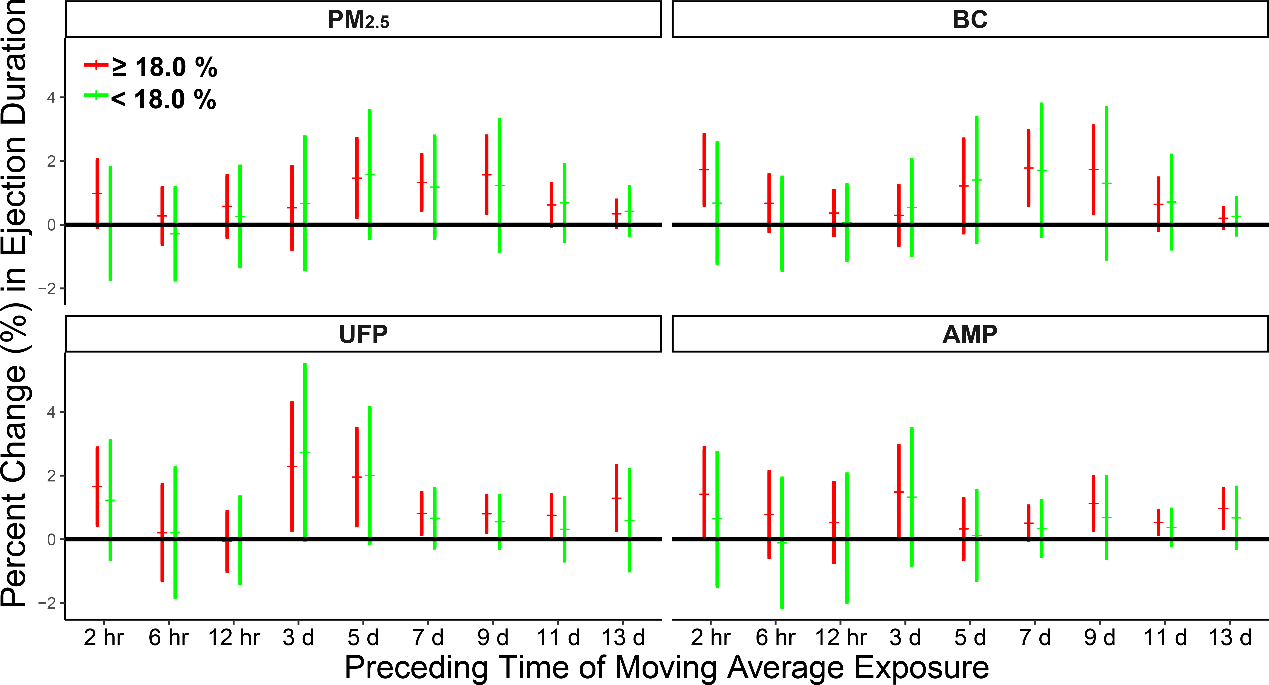


**Figure** **S27** **percent changes in ejection duration associated with the IQR increases in concentrations of PM_2.5_ and its constituents stratified by BFR**. All models are adjusted for ambient temperature and relative humidity. The red and green error bars denote the estimated changes in subjects with BFR ≥ and < 18.0 %, respectively. ^★^ Denotes significant (*p*-value < 0.05) differences between the estimates.


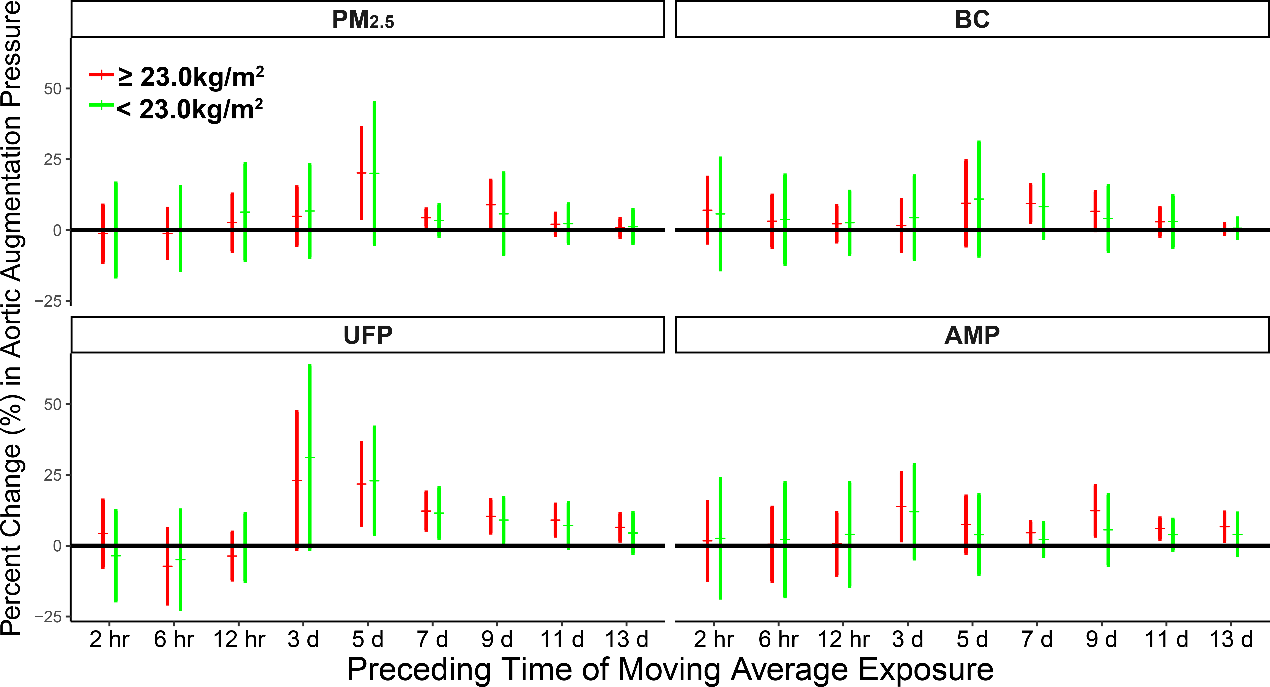


**Figure** **S28** **percent changes in aortic augmentation pressure associated with the IQR increases in concentrations of PM_2.5_ and its constituents stratified by BMI**. All models are adjusted for ambient temperature and relative humidity. The red and green error bars denote the estimated changes in subjects with BMI ≥ and < 23.0 kg/m^2^, respectively. ^★^ Denotes significant (*p*-value < 0.05) differences between the estimates.


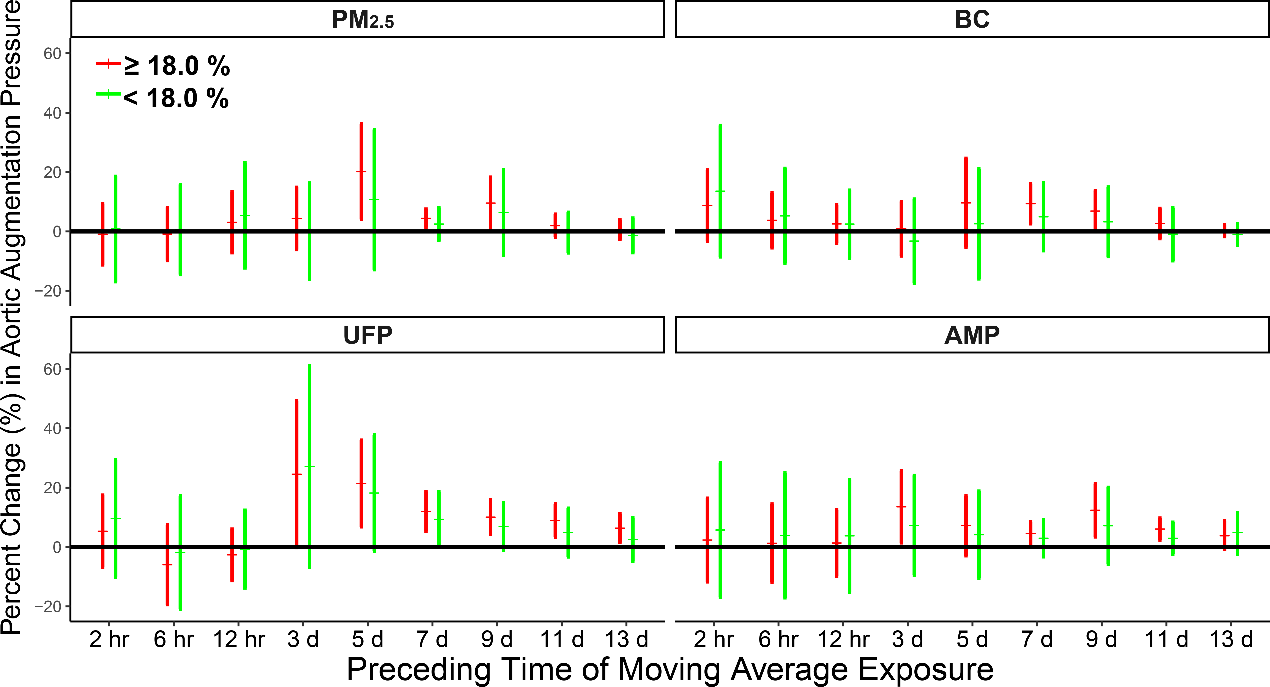


**Figure** **S29** **percent changes in aortic augmentation pressure associated with the IQR increases in concentrations of PM_2.5_ and its constituents stratified by BFR**. All models are adjusted for ambient temperature and relative humidity. The red and green error bars denote the estimated changes in subjects with BFR ≥ and < 18.0 %, respectively. ^★^ Denotes significant (*p*-value < 0.05) differences between the estimates.


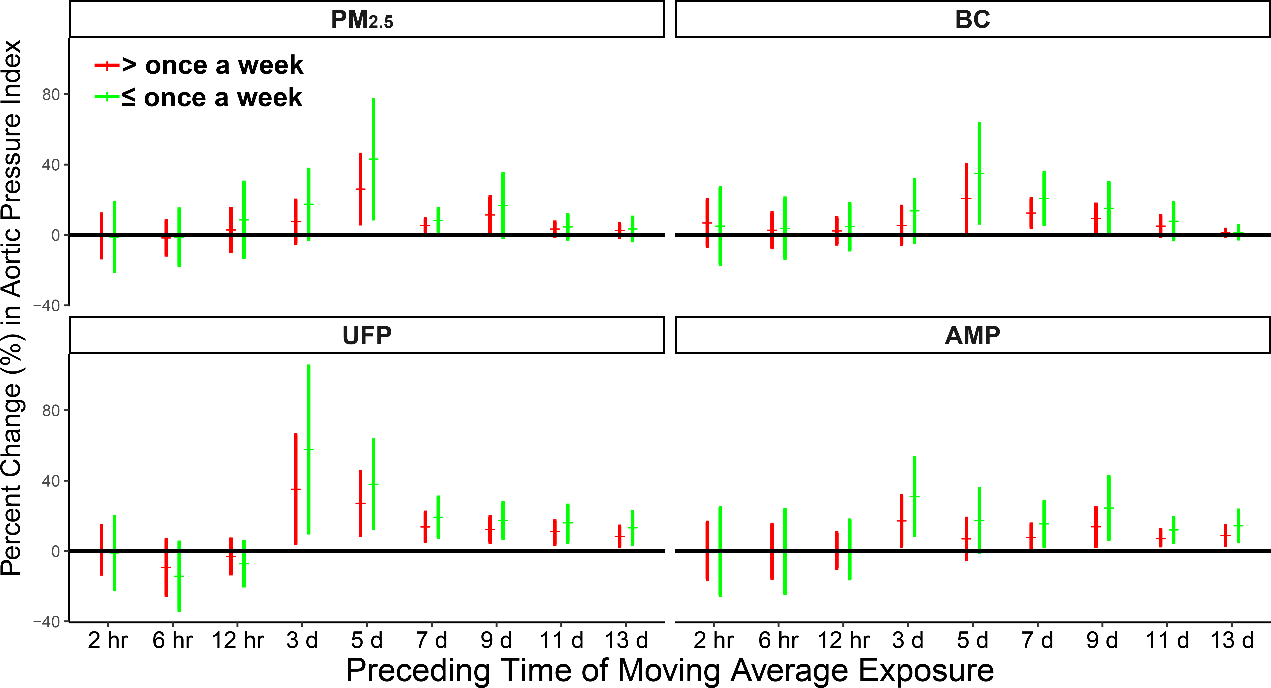


**Figure** **S30** **percent changes in aortic pressure index associated with the IQR increases in concentrations of PM_2.5_ and its constituents stratified by exercise frequency**. All models are adjusted for ambient temperature and relative humidity. The red and green error bars denote the estimated changes in subjects who exercise more and no more than once a week, respectively. ^★^ Denotes significant (*p*-value < 0.05) differences between the estimates.


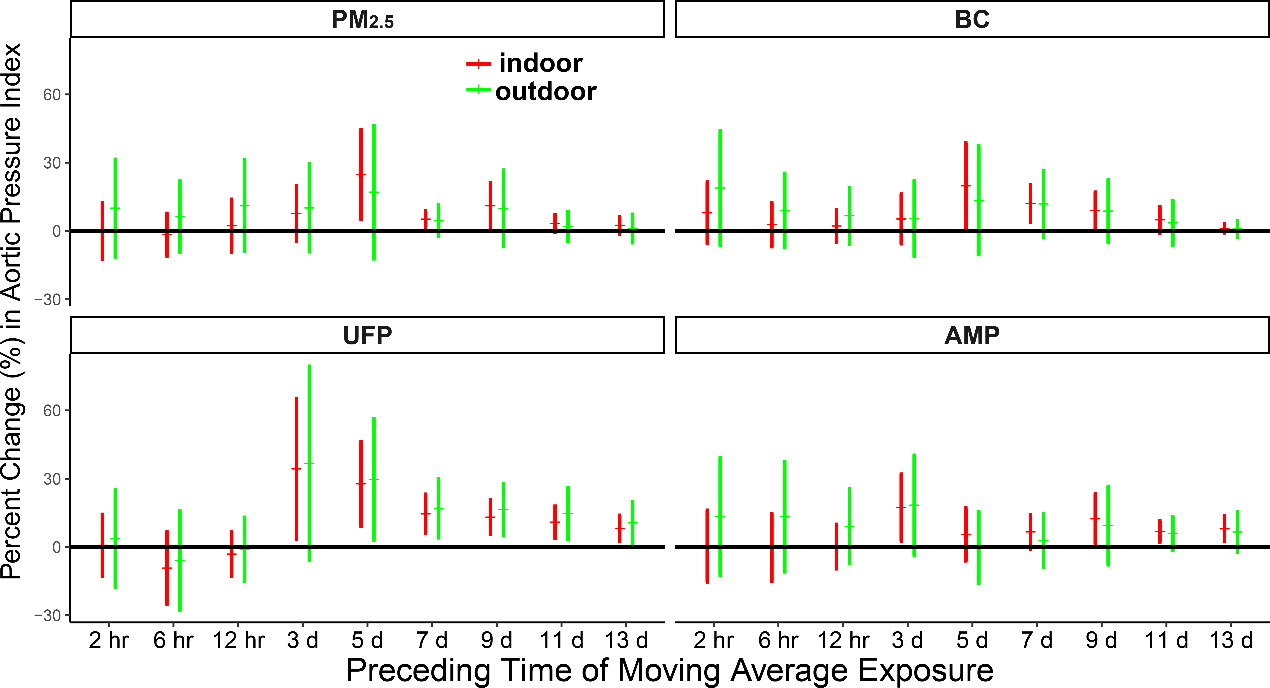


**Figure** **S31** **percent changes in aortic pressure index associated with the IQR increases in concentrations of PM_2.5_ and its constituents stratified by exercise place**. All models are adjusted for ambient temperature and relative humidity. The red and green error bars denote the estimated changes in subjects who prefer indoor and outdoor exercise, respectively. ^★^ Denotes significant (*p*-value < 0.05) differences between the estimates.


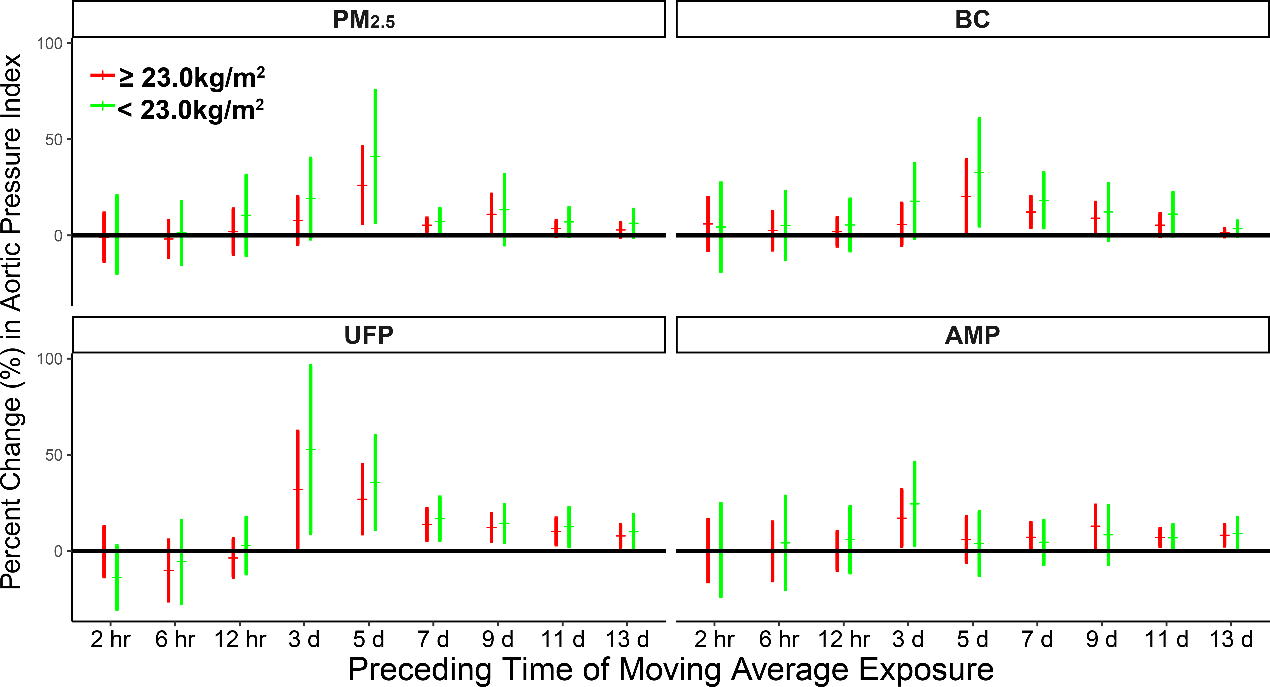


**Figure** **S32** **percent changes in aortic pressure index associated with the IQR increases in concentrations of PM_2.5_ and its constituents stratified by BMI**. All models are adjusted for ambient temperature and relative humidity. The red and green error bars denote the estimated changes in subjects with BMI ≥ and < 23.0 kg/m^2^, respectively. ^★^ Denotes significant (*p*-value < 0.05) differences between the estimates.


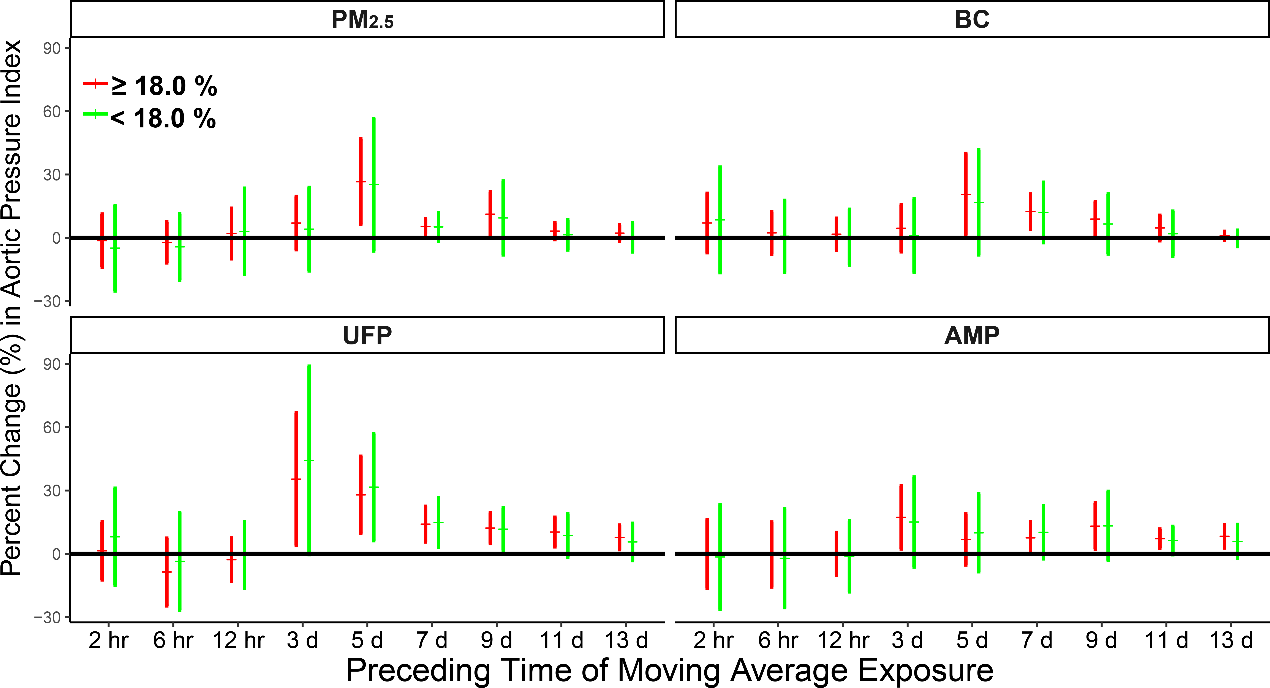


**Figure** **S33** **percent changes in aortic pressure index associated with the IQR increases in concentrations of PM_2.5_ and its constituents stratified by BFR**. All models are adjusted for ambient temperature and relative humidity. The red and green error bars denote the estimated changes in subjects with BFR ≥ and < 18.0 %, respectively. ^★^ Denotes significant (*p*-value < 0.05) differences between the estimates.
